# Cardiomyocyte Foxp1‐Specific Deletion Promotes Post‐injury Heart Regeneration via Targeting Usp20‐HIF1ɑ‐Hand1 Signaling Pathway

**DOI:** 10.1002/advs.202412124

**Published:** 2025-02-03

**Authors:** Yanfang Wang, Xiaoyu Wang, Ji Fang, Xiaoli Chen, Teng Xu, Tao Zhuang, Sheng Peng, Wenzhen Bao, Wenrun Wu, Yushi Lu, Haikun Wang, Brian Tomlinson, Paul Chan, Shougang Zhuang, Qi Zhang, Lin Zhang, Zhongmin Liu, Jingjiang Pi, Yuzhen Zhang, Jie Liu

**Affiliations:** ^1^ State Key Laboratory of Cardiovascular Diseases and Medical Innovation Center Shanghai Heart Failure Research Center Department of Cardiovascular Surgery Shanghai East Hospital Tongji University School of Medicine Shanghai 200120 China; ^2^ Department of Cardiology The First Affiliated Hospital of Wannan Medical College (Yijishan Hospital of Wannan Medical College) Wuhu Anhui 241001 China; ^3^ Institute of Translational Medicine Baotou Central Hospital Baotou Inner Mongolia 014040 China; ^4^ Department of Physiology and Pathophysiology School of Basic Medical Sciences Fudan University Shanghai 200032 China; ^5^ Department of Trauma Shanghai East Hospital Tongji University School of Medicine Shanghai 200120 China; ^6^ Faculty of Medicine Macau University of Science and Technology Macau SAR 999078 China; ^7^ Division of Cardiology Department of Internal Medicine Wan Fang Hospital Taipei Medical University Taipei Taiwan 11696 China; ^8^ Depeartment of Nephrology Shanghai East Hospital Shanghai 200120 China; ^9^ Department of Medicine Rhode Island Hospital and Warren Alpert Medical School of Brown University Providence Rhode Island USA; ^10^ Shenzhen Ruipuxun Academy for Stem Cell and Regenerative Medicine Shenzhen Guangdong 518122 China

**Keywords:** forkhead box P1, heart regenerations, metabolic transitions, myocardial infarctions

## Abstract

The adult mammalian heart has limited regenerative capacity to replace lost tissue after a major injury. Forkhead box P1 (Foxp1) regulates embryonic cardiomyocyte proliferation and heart development. However, whether Foxp1 participates in postnatal‐injury cardiomyocyte proliferation and heart regeneration remains unclear. This study demonstrates that Foxp1 is downregulated at border zone cardiomyocytes of both neonatal apical resection and adult myocardial infarction. Analysis of the Single‐cell transcriptome database reveals reduced Foxp1 expression in the cardiomyocyte population with high regenerative capacity. Cardiomyocyte‐Foxp1 loss‐of‐function significantly promotes, whereas cardiomyocytes‐Foxp1 gain‐of‐function suppresses cardiomyocyte proliferation. Mechanistically, Foxp1 directly regulates ubiquitin specific peptidase 20 (USP20), a de‐ubiquitinase that prevents hypoxia inducible factor 1ɑ (HIF1α) degradation. Thus, Foxp1 regulates HIF1α and downstream heart and neural crest derivatives expressed 1 (Hand1) to control the cardiomyocyte proliferation via metabolic transition from fatty acid oxidation to glycolysis. Finally, cardiac type troponin T2 (cTnT)‐promoter‐driven adeno‐associated virus 9 (AAV9) for Hand1 induction in cardiomyocytes significantly promoted cardiac regeneration and functional recovery. These findings may provide novel molecular strategies to promote heart regeneration and therapeutic interventions for heart failure.

## Graphical abstract

This study shows that Foxp1 regulates the USP20–HIF1ɑ‐Hand1 pathway, controlling cardiomyocyte metabolic reprogramming, proliferation, and heart regeneration. By targeting Hand1, the downstream effector, this work suggests a promising molecular target for gene therapy of heart failure, potentially guiding the development of novel molecular interventions to enhance cardiac repair.

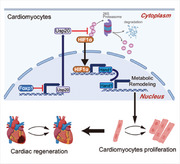

## 1 Introduction

Cardiovascular diseases are the leading cause of death worldwide, whilst the definitive treatment remains a major unmet clinical need.^[^ ^1^ ^]^ It has been well documented that the adult mammalian heart has limited regenerative capacity and large injuries of the adult heart lead to loss of cardiomyocytes (CMs), which is often followed by scar formation, resulting in a progressive deterioration of the heart contractile capacity, ultimately leading to end‐stage heart failure. Given the lack of therapeutic approaches to reverse the loss of functional myocardium, the development of efficient and safe regenerative procedures represents an urgent need in the field of modern cardiovascular research.

Emerging evidence supports the notion that new CMs are continuously born in the adult mammalian heart in homeostasis, although controversial published data regarding the possible sources and generation rates of these cells are a matter of scientific debate.^[^ ^2^, ^3^ ^]^ Recently, it has become clear that the mammalian heart is not a post‐mitotic organ. In fact, newborn mammals, such as one‐day‐old pigs ^[^ ^4^, ^5^ ^]^ and mice,^[^ ^6^, ^7^ ^]^ possess the capacity to completely regenerate the myocardium through notable levels of CMs proliferation, although this mechanism is markedly diminished after the first week of life.

Myocardial infarction (MI) border zone proliferating CMs were recently implicated as a source of heart regeneration in both zebrafish ^[^ ^8^ ^]^ and mammals.^[^ ^9^ ^]^ These CMs have very distinct transcriptomes, with reduced mitochondrial gene expression, reduced mitochondrial activity, increased glycolytic gene expression, and increased glucose uptake that resemble the proliferating embryonic CMs.^[^ ^8^ ^]^ Signaling networks that drive embryonic heart development may control specific mechanisms of postnatal heart regeneration. Therefore, one promising approach to improve the prognosis of heart failure is to extend this endogenous embryonic and neonatal cardiac regenerative capability to adult hearts. Understanding these mechanisms is of crucial importance for the development of novel therapeutic targets for CMs proliferation to facilitate cardiac regeneration and repair.

Forkhead box P1 (Foxp1), a large modular transcription repressor that binds to DNA via its highly conserved DNA‐binding domain, encodes a transcription factor important for the early development of many organ systems.^[^ ^10^, ^11^ ^]^ Myocardial‐specific loss of Foxp1 during embryonic heart development significantly increased CMs proliferation in mice,^[^ ^12^ ^]^ and haploinsufficiency of FOXP1 was associated with human congenital heart defects (CHDs) with the orthologous murine Foxp1 p.Pro596Ser mutant protein displaying elevated NK2 homeobox 5 (Nkx2.5) expression in cardiomyoblasts to promote proliferation.^[^ ^13^ ^]^ However, whether Foxp1 plays an important role in the adult heart in response to injury and how Foxp1 impacts cardiac regeneration and repair, remains elusive.

Here, we demonstrated that Foxp1 regulates CMs proliferation following neonatal apical resection (AR) and adult MI to control heart regeneration and repair. We identified the ubiquitin specific peptidase 20 (Usp20), which de‐ubiquitinates hypoxia‐inducible factor 1ɑ (HIF1ɑ) and prevents it from proteasomal degradation, as Foxp1 direct target gene. Deletion of Foxp1 in CMs awakes a dormant regenerative program in neonatal and adult mice via a HIF1α‐dependent control of heart and neural crest derivatives expressed 1 (Hand1) expression that, in turn, regulates the transition from fatty acid (FA) oxidation to glycolysis in CMs. Finally, a cardiac type troponin T2 (cTnT)‐promoter‐driven Hand1‐adeno‐associated virus 9 (AAV9) significantly attenuated the Foxp1 gain‐of‐function in CMs which triggered a reduction of CMs proliferation and thus suppressed impaired cardiac regeneration, repair, and cardiac function. Altogether, we propose that the potential of endogenous regenerative properties in adult hearts by the Foxp1‐Usp20‐HIF1ɑ‐Hand1 signaling pathway might represent a future promising therapeutic approach for heart failure.

## 2 Results

### 2.1 Loss of Cardiomyocyte‐Foxp1 Increases Cell Proliferation and Promotes Heart Regeneration in the Neonatal Apical Resection Model

**Figure 1:**
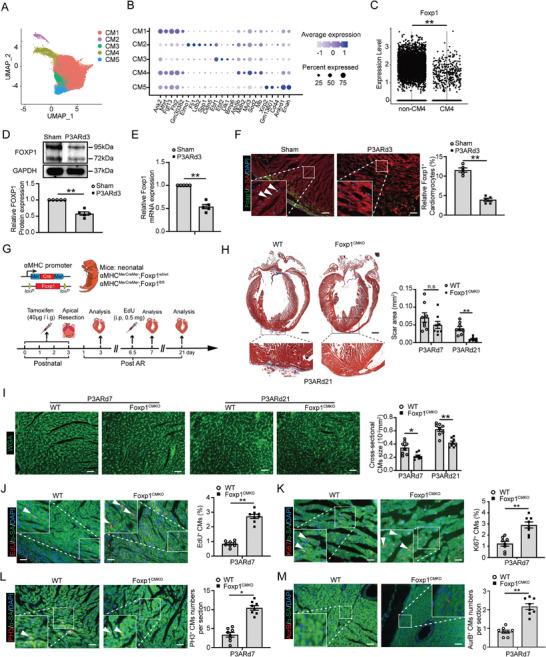
Loss of Foxp1 in cardiomyocytes increases cell proliferation and promotes heart regeneration in the neonatal apical resection model. A) Uniform manifold approximation and projection (UMAP) visualization of cardiomyocyte clusters colored by identity in the neonatal heart of single nucleus RNA sequencing datasets GSE130699. B) Dot plot of the expression of gene signatures of cardiomyocyte CM1‐CM5 populations according to Cui et al.,^[^ ^14^ ^]^ C) Violin plots of Foxp1 expression in high regenerative capacity immature cardiomyocytes (regenerative) and the other non‐regenerative mature CMs (non‐regenerative) of neonatal‐MI hearts. D–F) Foxp1 expression in CMs of hearts 3 days after apical resection (AR) or sham‐operated at postnatal day 3 (P3ARd3) by D) western blot, E) RT‐qPCR, and co‐immunostaining of Foxp1 and ɑ‐sacromeric actinin (ɑ‐SA) of F) heart sections (*n =* 5). G) Schematic diagram of generation of CM‐specific Foxp1 deletion mice (Foxp1^CMKO^) for neonatal cardiac regeneration post‐AR, and administration of tamoxifen (40 µg per day, from postnatal day 0 to 3) to induce Foxp1 deletion in CMs for AR operation, with EdU incorporation quantification for CM proliferation, histology analysis for final cardiac morphology, and echo analysis for cardiac function. H,I) Masson trichrome staining of fibrotic scar area (H) and wheat germ agglutinin (WGA) staining of myocyte cross–sectional size (I) in P3ARd7 and P3ARd21 hearts of Foxp^CMKO^ mice and wild‐type littermates. The representative images are on the left and the quantification data right (*n =* 8). J–M) CM proliferation quantified by J) EdU incorporation, K) immunostaining of Ki67 (cell cycles), L) PH3 (karyokinesis), and M) Aurora B (mitosis) of ɑ‐SA^+^ CMs in P3ARd7 hearts of Foxp^CMKO^ mice and wild‐type littermates. The representative images are on the left and the quantification data right (*n =* 8). Data are means ± SEM. *p < 0.05; ** *p* < 0.01, n.s. indicates not significant. Scale bar: (F) and (I) through (M), 50 µm; (H), 2 mm.

Recent single cell (sc)‐RNA sequencing of murine neonatal myocardial infarction (MI) hearts revealed crucial regulatory networks governing heart regeneration.^[^ ^14^ ^]^ Therefore we analyzed the data with accession No. GSE130699.^[^ ^14^ ^]^ This single nucleus RNA‐sequencing dataset identified a unique immature CMs population enriched with genes of the immature heart (*Tnni*, *Myh7*, and *Actc1*), cell‐cycle progression (*Aurka*, *Ccnb1*, *Ki67*, *Cdk1*, and *Cdk4*), anti‐oxidant genes (*Prdx1*, *Sod1*, *Sod2*, and *Cat*), glycolysis (*Tpi1* and *Aldoa*) and reduced maturation genes (*Myh6*, *Ryr2* and *Cacna1c*). Importantly, the high regenerative capacity immature neonatal CMs population showed a significantly reduced Foxp1 expression compared with other mature CMs populations (**Figure** **1**A–C). Furthermore, we confirmed significantly reduced Foxp1 expression in neonatal apical resection (AR) hearts (Figure 1D,E), and immunostaining demonstrated reduced expression mainly in border zone CMs (Figure 1F). These results suggest a very likely pivotal role for Foxp1 in CMs in the regulation of cardiac regeneration and repair after injury.

To determine the Foxp1 effect in vivo, we generated mice with specific deletion of Foxp1 in CMs (Foxp1^CMKO^). Tamoxifen administration was performed from postnatal day 0 to day 3 (Figure 1G). Foxp1 expression was specifically lost in CMs of Foxp1^CMKO^ mutant mice as validated by reverse transcription quantitative real‐time polymerase chain reaction (RT‐qPCR), western blot, and immunostaining (Figure S1A–E, Supporting Information). Interestingly, neither appreciable changes in heart size or gross morphology (Figure S1F, Supporting Information), nor cardiac function by echocardiography (Table S1, Supporting Information) was observed between Foxp1^CMKO^ mice and wild‐type littermates up to 12 weeks of age. Next, we examined the heart after AR at postnatal 3 days (P3AR). Foxp1^CMKO^ mice exhibited significantly less scar tissue formation in post‐AR 7 days (P3ARd7) and 21 days (P3ARd21) (Figure 1H) as well as smaller border zone CMs (Figure 1I) compared with wild‐type littermates, indicating that Foxp1^CMKO^ might have increased cardiac regenerative capacity.

We then examined CMs proliferation at P3ARd7 and Foxp1^CMKO^ exhibited significantly elevated CMs proliferation compared with wild‐type littermates as quantified by 5‐ethynyl‐2′deoxyuridine (5‐EdU) incorporation (>3.5 fold) (Figure 1J), active cell cycle phases (G1, S, G2, and M) marker Ki67 (>3 fold) (Figure 1K), karyokinesis marker phosphorylated histone H3 (PH3) (>3 fold) (Figure 1L) and mitosis marker Aurora B kinase in the cleavage furrow (>2.5 fold) (Figure 1M) co‐stained with the specific CMs marker, α‐sarcomeric actinin (α‐SA). For better visualization of the cardiomyocytes, we co‐stained these proliferation markers with cardiac nuclear specific pericentriolar material 1 (Pcm1), which showed similar elevated proliferating CMs in Foxp1^CMKO^ compared with wildtype littermates (Figure S1G–I, Supporting Information).

Concomitant with the in vivo study, Foxp1 knockdown in cultured neonatal mouse cardiomyocytes (NMCMs) (Figure S2A,B, Supporting Information) triggered increased cell proliferation, as evidenced by 5‐EdU incorporation, PH3, and Aurora B staining (Figure S2C–E, Supporting Information). This was further supported by the upregulation of cell cycle genes (Figure S2F,G, Supporting Information).

Altogether, these results indicated that ablation of Foxp1 in CMs results in a significant increase of neonatal CMs proliferation leading to enhanced cardiac regeneration capacity, thereby contributing to improved post‐injury tissue repair with less scar formation in the neonatal AR model.

### 2.2 Loss of Cardiomyocyte‐Foxp1 Increases Cell Proliferation to Promote Heart Regeneration And Improve Cardiac Function in the Adult Myocardial Infarction Model

**Figure 2:**
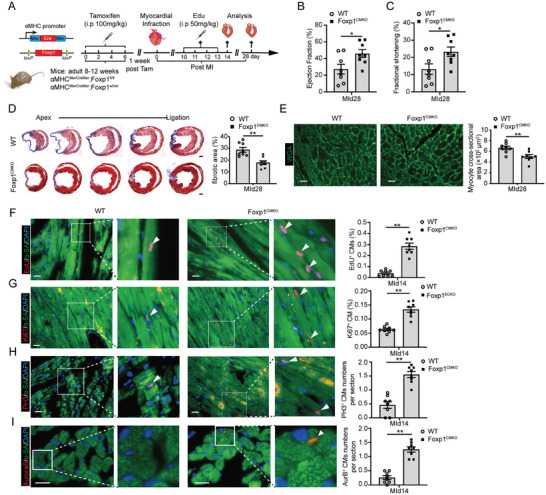
Loss of Foxp1 in cardiomyocytes increases cell proliferation to promote heart regeneration and improve cardiac function in the adult myocardial infarction model. A) Schematic diagram of adult cardiac regeneration post‐myocardial infarction (MI), and administration of tamoxifen (100 mg k^−1^g, ip, every other day for a total 4 injections) for the induction of Foxp1 deletion in CMs for MI operation, with EdU incorporation quantification for CM proliferation, histology analysis for final cardiac morphology and echo evaluation for cardiac function. B,C) Echocardiography parameters left ventricle ejection fraction (B) and fractional shortening C) at post‐MI 28 days (MId28) of Foxp1^CMKO^ and wild‐type littermates (*n =* 8). D,E) Masson trichrome staining of fibrotic scar area D) and WGA staining of cross‐sectional size of border zone CMs (E) at MId28 of Foxp1^CMKO^ and wild‐type littermates. The representative images are on the left and the quantification data right (*n =* 8). F–I) Border zone CM proliferation in MId14 hearts from Foxp^CMKO^ mice and wild‐type littermates quantified by F) EdU incorporation, G) co‐immunostaining of Ki67, H) PH3 and I) Aurora B with ɑ‐SA. The representative images are on the left and the quantification data right (*n =* 8). Data are means ± SEM. *p < 0.05; ** *p* < 0.01, n.s. indicates not significant. Scale bar: (D), 2 mm; (E) through (I), 50 µm.

We examined whether Foxp1 signaling plays a similar promoting effect in adult heart regeneration. Tamoxifen was administered to induce Foxp1 deletion in CMs in mice aged 8–12 weeks, and MI was performed by permanent ligation of the proximal left anterior descending coronary artery (**Figure** **2**A; Figure S3A,B, Supporting Information). We found no significant difference in infarct size or area at risk between Foxp1^CMKO^ and wild‐type littermates 1‐day post‐MI (MId1) (Figure S3C, Supporting Information). Echocardiography showed normal heart size and function in all sham‐operated animals; however, Foxp1^CMKO^ mice exhibited significantly improved cardiac function, evidenced by increased left ventricular ejection fraction (LVEF) and fractional shortening (FS) (Figure 2B,C; Table S2, Supporting Information), as well as reduced fibrotic scar size (Figure 2D) and border zone CMs size (Figure 2E) compared with wild‐type littermates. Moreover, we observed a gradually improved cardiac function and survival in Foxp1^CMKO^ mice by long‐term observation (Figure S3D,E, Supporting Information). These results indicate that specific deletion of Foxp1 in CMs improves cardiac repair and function in the adult MI model.

There is limited CM mitosis in adult hearts although sustained induction of CM cell cycle from birth by overexpression of cell cycle regulators has been achieved.^[^ ^15^, ^16^ ^]^ However, cell cycle re‐entry in adult CMs appears restricted to a mono‐nucleated subpopulation. ^[^ ^17^ ^]^ We isolated adult mouse CMs by collagenase digestion and found a significant increase in mononucleated CMs percentage with the decrease of bi‐nucleated CMs percentage at the border zone of MId7 hearts in Foxp1^CMKO^ mice (Figure S3F, Supporting Information). Immunostaining of heart sections revealed increased mitotic CMs, detected by elevated EdU incorporation (>6 fold) (Figure 2F; Figure S3G, Supporting Information), Ki67^+^ (>2.8 fold) (Figure 2G; Figure S3H, Supporting Information), PH3^+^ (>2.5 fold) (Figure 2H; Figure S3I, Supporting Information), and Aurora B^+^ (>2 fold) (Figure 2I) at MId14 in Foxp1^CMKO^ compared with wild‐type littermates.

To further validate whether Foxp1 deletion promotes cell proliferation in human cardiomyocytes, we used human induced pluripotent stem cell‐derived cardiomyocytes (iPSC‐CMs). Foxp1‐siRNA knockdown significantly promotes iPSC‐CMs proliferation, as shown by 5‐EdU incorporation and PH3 staining (Figure S3J–M, Supporting Information).

These results demonstrated that Foxp1 regulates adult CM mitotic nucleation status and cell proliferation, promoting a proliferative mononuclear CM population in Foxp1‐deletion mutant hearts. This mechanism contributes to enhancing heart regeneration and facilitates cardiac repair following MI in adults.

### 2.3 Foxp1 Gain‐Of‐Function In Cardiomyocytes Suppresses Proliferation To Impairs Heart Regeneration And Function

**Figure 3:**
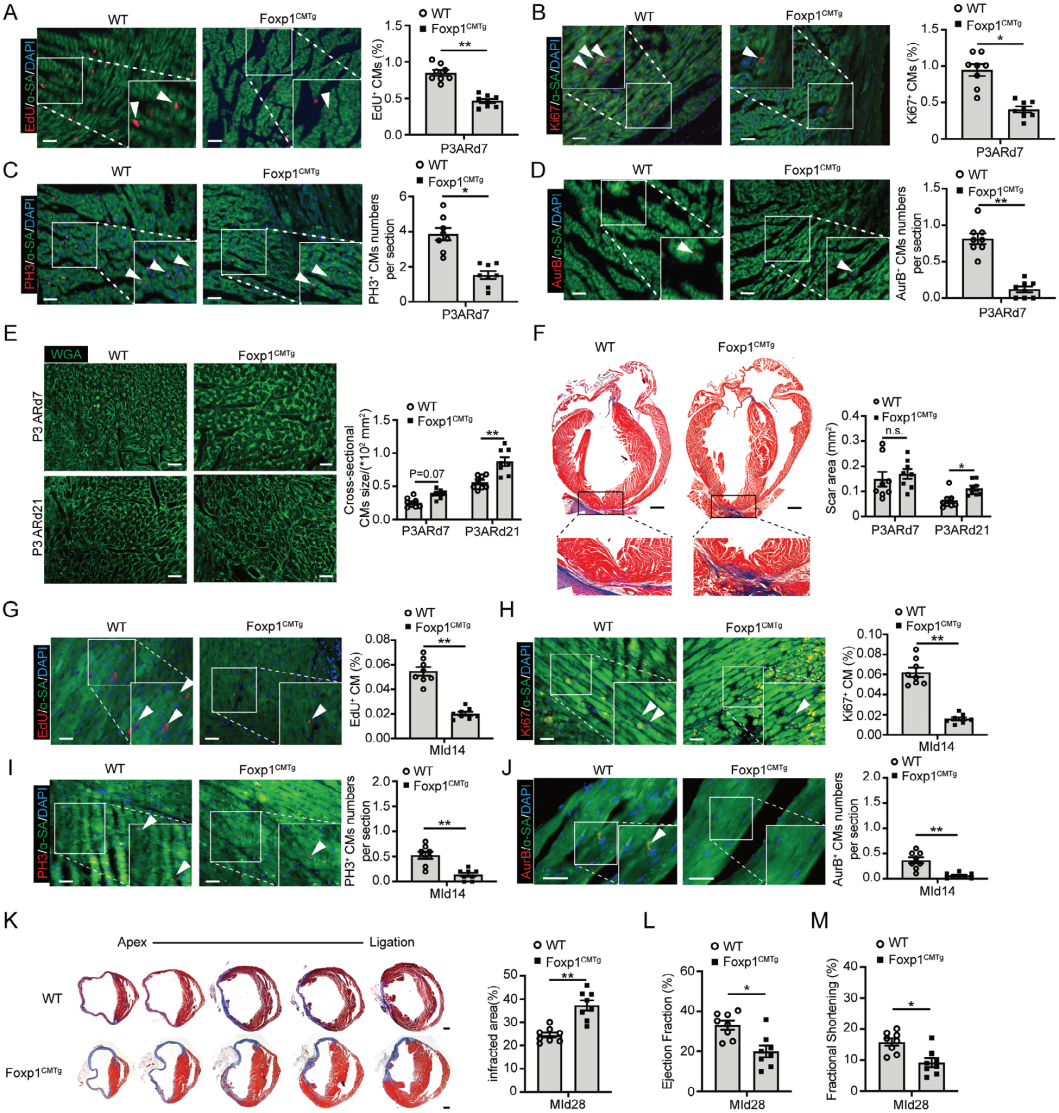
Foxp1 gain‐of‐function in cardiomyocytes suppresses proliferation and impairs heart regeneration and function. A–D) CM proliferation quantified by A) EdU incorporation, B) immunostaining of Ki67, C) PH3, and D) Aurora B in P3ARd7 hearts of Foxp^CMTg^ mice and wild‐type littermates, with representative images on the left and quantification data right (*n =* 8). E–J) WGA staining of myocyte cross‐sectional size E) and Masson trichrome staining of fibrotic scar area F) in P3ARd7 and P3ARd21 hearts, and border zone CM proliferation quantified by EdU incorporation G), immunostaining of Ki67 H), PH3 I) and Aurora B J) in MId14 hearts from Foxp^CMTg^ mice and wild‐type littermates. The representative images are on the left and the quantification data right (*n =* 8). K‐M, Masson trichrome staining of cardiac fibrotic scar area K), and echocardiography parameters left ventricle ejection fraction L) and fractional shortening M) evaluation of cardiac function at MId28 of Foxp1^CMKO^ mice and wild‐type littermates. The representative images are on the left and the quantification data right (*n =* 8). Data are means ± SEM. *P<0.05; ** *P*<0.01, n.s. indicates not significant. Scale bar: (A) through (E) and (G) through (J), 50 µm; (F) and (K), 2 mm.

In contrast to Foxp1 deletion in CMs, Foxp1 gain‐of‐function using transgenic Foxp1^CMTg^ mice (Figure S4A–C, Supporting Information) triggered a statistically significant reduced capacity of heart regeneration in neonatal AR and adult MI models. Significantly reduced mitotic CMs in P3ARd7 hearts were observed by the decreased percentage of proliferating CMs in Foxp1^CMTg^ mice compared with wild‐type littermates (**Figure** **3**A–D). Further studies showed increased border zone CMs size (Figure 3E) and fibrotic scar size (Figure 3F) of P3ARd7 and P3ARd21 hearts in Foxp1^CMTg^ mice, suggesting that Foxp1‐induced expression in CMs worsens the regeneration capacity of neonatal AR hearts.

Similarly reduced proliferation in CMs was observed at the border zone of adult MI hearts (Figure 3G–J), and greater fibrotic scar size (Figure 3K) as well as deterioration of cardiac function (Figure 3L,M; Table S2, Supporting Information) in Foxp1^CMTg^ compared with wild‐type littermates, suggesting that CM‐Foxp1 induced expression attenuates CM regenerative capability and deteriorates cardiac function in adult MI model.

### 2.4 HIF1ɑ Deletion in Cardiomyocytes Reverses the Foxp1‐Dependent Elevation Of Cell Proliferation in Heart Regeneration

**Figure 4:**
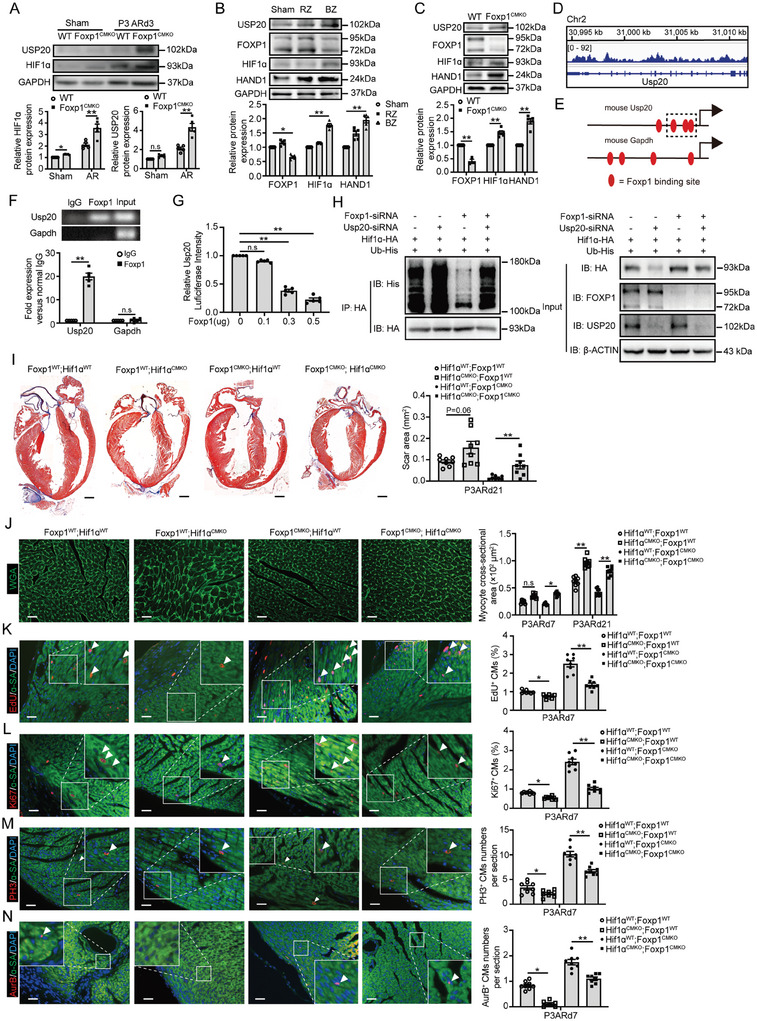
Hypoxia‐inducible factor 1ɑ (HIF1ɑ) deletion in cardiomyocytes reverses the Foxp1‐dependent elevation of cell proliferation in heart regeneration. A) HIF1ɑ and USP20 protein expression by western blot in CMs from P3ARd3 and sham‐operated hearts of Foxp1^CMKO^ mice and wild‐type littermates, with representative blots on the top and quantification data bottom (*n =* 5). B) The protein expression of FOXP1, HIF1ɑ, USP20, and HAND1 in CMs from border zone (BZ) and remote zone (RZ) of MId14 and sham‐operated hearts, with representative blots on the top and quantification data bottom (*n =* 5). C) The protein expression of FOXP1, HIF1ɑ, USP20, and HAND1 in border zone CMs from MId14 hearts of Foxp1^CMKO^ mice and wild‐type littermates, with representative blots on the top and quantification data bottom (*n =* 5). D) CUT&Tag assay showed Foxp1 enrichment in the promoter region of Usp20. E) Schematic diagram of Foxp1 binding sites in the proximal 6 kb promoter of Usp20, with amplified sequence indicated in the dashed box cloned into a vector for luciferase reporter assay. F) Chromatin immunoprecipitation (ChIP)‐qPCR of Foxp1 and the promoter of Usp20 in the bottom with agarose gel on the top (*n =* 5). G) Luciferase reporter assay of Foxp1 for Usp20 promoter in NIH‐3T3 cells (*n =* 5). H) HEK293T cells were transfected with Foxp1‐ or Usp20‐siRNA following co‐transfection of HIF1ɑ‐HA and Ubiquitin (Ub)‐His plasmid. The cell lysates were immunoprecipitated by anti‐HA antibody or and the precipitates were immunoblotted by an anti‐His antibody for observation of the effect on HIF1ɑ deubiquitylation. I,J) Masson trichrome staining of cardiac fibrotic scar area I), WGA staining of CM cross‐sectional area J) in P3ARd7 and P3ARd21 hearts of HIF1ɑ; Foxp1^CMKO^, Foxp1^CMKO^, HIF1ɑ^CMKO^ and wild‐type mice (*n =* 8). K–N) CM proliferation quantified by EdU incorporation K), immunostaining of Ki67 L), PH3 M), and Aurora B N) of ɑ‐SA^+^ CMs in P3ARd7 hearts of HIF1ɑ;Foxp1^CMKO^, Foxp1^CMKO^, HIF1ɑ^CMKO^, and wild‐type mice. The representative images on the left and the quantification data are on the right (*n =* 8). Data are means ± SEM. *p < 0.05; ** *p* < 0.01, n.s. indicates not significant. Scale bar: (I), 2 mm; (J) through (N), 50 µm.

A study using HIF1ɑ oxygen‐dependent degradation domain fused Cre recombinase estrogen receptor T2 (Cre^ERT2^) mice and identified a rare population of hypoxic CMs that display neonatal features of proliferative CMs of smaller size, mononucleation, and less oxidative DNA damage.^[^ ^18^ ^]^ A later report confirmed that adult mouse CMs could induce this neonatal proliferation state and thus stimulate heart regeneration when exposed to gradual systemic hypoxia.^[^ ^19^ ^]^ In the present work, we found a significantly elevated expression of HIF1ɑ and its degradation inhibition de‐ubiquitinase, USP20 in P3AR CMs and adult MI border zone CMs in Foxp1^CMKO^ mice (**Figure** **4**A–C), implying that Foxp1 might regulate HIF1ɑ expression in order to induce CM proliferation for heart regeneration.

HIF1α protein expression is usually regulated by the balance of von Hippel‐Lindau tumor suppressor (pVHL) ubiquitin E3 ligase for degradation and de‐ubiquitinase USP20 preventing degradation.^[^ ^20^ ^]^ Foxp1 is usually a transcription repressor by reducing gene expression via binding to the gene promoter region with the 5′‐TRTTKTY‐3′ sequence.^[^ ^10^ ^]^ Cleavage under targets and tagmentation (CUT&Tag) analysis showed that FOXP1 was enriched in promoter regions of Usp20 (Figure 4D) and sequence analysis elucidated FOXP1 binding sites in the promoter region of mouse Usp20 (Figure 4E). Chip‐qPCR showed an association of Foxp1 with the promoter of Usp20 (Figure 4F; Table S5, Supporting Information) and luciferase assay confirmed that Foxp1 expression vector could dose‐dependently repress the promoter of Usp20 (Figure 4G) containing FXOP1 binding sites in NIH‐3T3 cells. HIF1α ubiquitylation analysis by co‐immunoprecipitation showed that Foxp1‐siRNA knockdown significantly downregulates HIF1α ubiquitination and elevates HIF1α protein level as well, whereas Usp20‐siRNA knockdown reversed Foxp1‐siRNA induced HIF1α de‐ubiquitination and elevated protein levels (Figure 4H). These results overall suggest that Foxp1 regulates the de‐ubiquitinase USP20 to maintain HIF1ɑ stability and expression in CMs.

To confirm the regulation of Foxp1 in CMs in HIF1ɑ signaling and its role in CM proliferation for heart regeneration, we generated mice with specific double deletion of HIF1ɑ and Foxp1 specifically in CMs (HIF1ɑ^CMKO^; Foxp1^CMKO^). As expected, HIF1ɑ deletion in CMs (Figure S5A–C, Supporting Information) significantly reversed the reduced fibrotic scar size (Figure 4I) and CM size at the AR border zone (Figure 4J) in Foxp1^CMKO^ mice, with no appreciable changes in heart weight/body weight and cardiac function (Table S1, Supporting Information). Further examination showed a similar significant reversal of elevated percentages of proliferating CMs (Figure 4K–N) in Foxp1^CMKO^ mice.

Consistent with the in vivo study, we found that HIF1ɑ knockdown (Figure S5D,E, Supporting Information) reversed the elevated cell proliferation in Foxp1‐knockdown NMCMs (Figure S5F–H, Supporting Information).

Taken together, these studies demonstrated that Foxp1 regulates HIF1ɑ signaling and influences CM proliferation contributing to cardiac regeneration and repair after injury.

### 2.5 Cardiomyocyte‐derived Foxp1‐HIF1ɑ Further Regulates Hand1, a Target Gene that Controls Metabolic Transition in Cell Proliferation and Heart Regeneration

**Figure 5:**
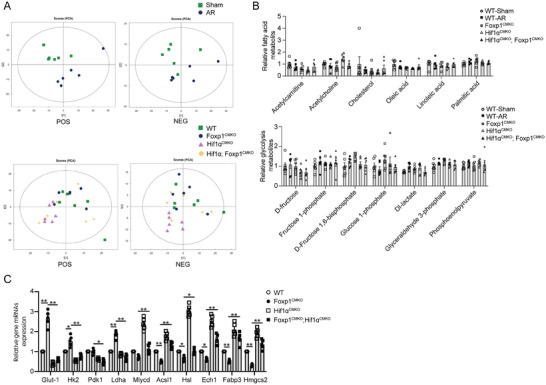
Cardiomyocytes‐derived Foxp1‐HIF1ɑ signaling regulates cardiac metabolic transition from fatty acid oxidation to glycolysis. A) Non‐targeted metabolomics of P3ARd7 or age‐matched sham‐operated hearts from Foxp1^CMKO^, HIF1ɑ^CMKO^, Foxp1; HIF1ɑ^CMKO^ and wild‐type littermates are performed, with the principal component analysis (PCA) indicating good separation of metabolite clusters between each group. B) Non‐targeted metabolomics results of elevated glycolysis and reduced fatty acid (FA) intermediates with differences of AR or sham‐operated wild‐type mice, or HIF1ɑ; Foxp1^CMKO^, Foxp1^CMKO^, HIF1ɑ^CMKO^ compared with wild‐type littermates (*n =* 6). C) The expression of key components of glycolysis genes (Glut‐1, Hk‐2, Pdk‐1, Ldha) and FA oxidation genes (*Mlycd, Acsl1, Hsl, Ech1, Fabp3* and *Hmgcs2*) by RT‐qPCR in the hearts of AR or sham‐operated wild‐type mice, or HIF1ɑ;Foxp1^CMKO^, Foxp1^CMKO^, HIF1ɑ^CMKO^ compared with wild‐type littermates (*n =* 5). Data are means± SEM. *p < 0.05; ** *p* < 0.01.

The metabolic transition from glycolysis to lipid oxidation occurs soon after birth and coincides with CM cell cycle arrest and regenerative capacity loss.^[^ ^21^, ^22^ ^]^ Moreover, the transition from fatty acid (FA) oxidation back to glycolysis is also important for CM proliferation and heart regeneration.^[^ ^23^ ^]^ To assess whether Foxp1 deletion in CMs mediates extensive proliferation of these cells in the AR heart model and is dependent on metabolic transition, we performed non‐targeted metabolomics. Principal component analysis (PCA) indicated a good separation of metabolite clusters between each group (**Figure** **5**A). Notably, the enrichment of glycolytic intermediates was observed, alongside the decline in the FA pathway intermediates in AR hearts compared with sham‐operated hearts (Figure 5B). Also, we found a significant upregulation of genes involved in glycolysis (Glut‐1, Hk‐2, Pdk‐1, Ldha) and downregulation of genes related to FA utilization and oxidation (Mlycd, Acsl1, Hsl, Ech1, Fabp3 and Hmgcs2) of p3ARd7 hearts (Figure 5C), suggesting that AR hearts have elevated glycolysis and reduced FA metabolism, and thus a metabolic transition facilitating CM proliferation for heart regeneration. Moreover, Foxp1^CMKO^ mice exhibited significantly elevated glycolysis and suppressed FA oxidation in p3ARd7 CMs compared with those of wild‐type littermates, while HIF1ɑ ablation in CMs significantly reversed the effect of Foxp1 deletion in CMs in increasing metabolic transition to glycolysis (Figure 5B,C). These results demonstrated a crucial role of the Foxp1‐HIF1ɑ signaling pathway in CMs in the regulation of metabolic transition responsible for cell proliferation and heart regeneration in the neonatal heart AR model.

**Figure 6:**
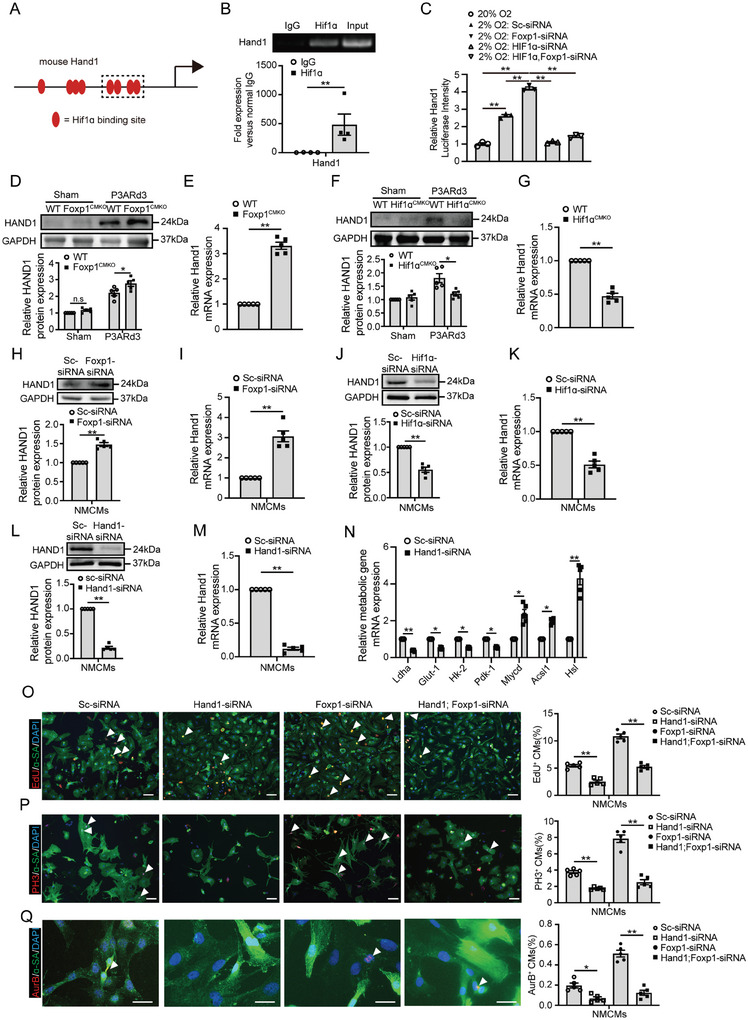
Cardiomyocytes‐derived Foxp1‐HIF1ɑ further regulate Hand1, a target gene that controls metabolic transition in cell proliferation and heart regeneration. A) Schematic diagram of HIF1ɑ binding sites within the proximal 3 kb promoter region of Hand1. The dashed box indicates the amplified sequence that was cloned into a luciferase reporter vector. B) Chromatin immunoprecipitation (ChIP)‐qPCR of HIF1ɑ binding to the Hand1 promoter in neonatal cardiomyocytes under 2% oxygen hypoxic conditions. The agarose gel image is shown above and the qPCR results are shown below (*n =* 4). C) Luciferase reporter assay for Hand1 promoter activity in NIH‐3T3 cells under the indicated treatments (*n =* 3). D–G) Hand1 expression by western blot and RT‐qPCR in CMs from P3ARd3 or sham‐operated hearts of Foxp1^CMKO^ (D‐E), HIF1ɑ^CMKO^ mice F–G) compared with their wild‐type littermates (*n =* 5). H–K) Hand1 expression by western blot and RT‐qPCR in neonatal mouse CMs (NMCMs) treated with Foxp1‐siRNA (H‐I), HIF1ɑ‐siRNA (J‐K) compared with scramble‐siRNA (*n =* 5). L,M) Hand1‐siRNA knockdown efficacy in NMCMs by western blot L) and RT‐qPCR M) (*n =* 5). N) The expression of lipid metabolizing and glycolysis genes in Hand1 knockdown or scramble‐siRNA CMs (*n =* 5). O‐Q, Cell proliferation quantified by EdU incorporation O), immunostaining of PH3 P) and Aurora B Q) in NMCMs treated by knockdown with Hand1‐siRNA; Foxp1‐siRNA, Foxp1‐siRNA, Hand1‐siRNA compared with scramble siRNA control (*n =* 5). Data are means± SEM. *p < 0.05; ** *p* < 0.01, n.s. indicates not significant. Scalebar: (O) through (Q), 50 µm.

Basic helix‐loop‐helix transcription factor Hand1 is highly expressed in the fetal heart under direct control of HIF1ɑ. Previous studies have shown that HIF1ɑ regulates Hand1, leading to the inhibition of lipid metabolism and promotion of glycolysis, eventually resulting in increased CM proliferation.^[^ ^24^ ^]^ In this study, we identified multiple HIF1ɑ binding sites within the proximal promoter region of Hand1 by sequence analysis (**Figure** **6**A). ChIP‐qPCR indicated that HIF1ɑ directly binds to the Hand1 promoter region in NMCMs under hypoxic conductions (Figure 6B). Luciferase reporter assay showed that the proximal Hand1 promoter containing HIF1ɑ binding sites was activated under reduced oxygen levels. This activation was further enhanced by Foxp1 knockdown and significantly reversed upon HIF1ɑ‐siRNA knockdown (Figure 6C). Additionally, a significantly elevated expression of Hand1 in P3ARd3 CMs of Foxp1^CMKO^ mice (Figure 6D,E), while reduction in P3ARd3 CMs of HIF1ɑ^CMKO^ mice (Figure 6F,G). HAND1 expression levels varied in parallel with reduced FOXP1 and elevated HIF1ɑ expression levels in border zone CMs of Foxp1^CMKO^ mice (Figure 4B,C). Concomitantly, siRNA knockdown of Foxp1 in cultured NMCMs induced a significant increase in Hand1 expression (Figure 6H,I) while HIF1ɑ knockdown induced decreased Hand1 expression (Figure 6J,K).

To further define the function of the Foxp1‐HIF1ɑ‐Hand1 signal pathway for metabolic transition, Hand1‐siRNA knockdown was performed in NMCMs (Figure 6L,M). Hand1 depletion in Foxp1 knockdown NMCMs significantly reversed the upregulation of glycolysis‐related genes and downregulation of lipid metabolizing genes (Figure 6N) contributing to the reversal of elevated proliferation (Figure 6O–Q). Moreover, the glucose consumption and lactate production assays demonstrated that glycolysis was more active when Foxp1 was knocked down in NMCMs. Furthermore, Hand1 or HIF1ɑ knockdown in NMCMs significantly reversed the glycolysis activation (Figure S6, Supporting Information) and thus reduced cell proliferation.

Collectively, these results demonstrated the major contribution of metabolic transition regulated by the Foxp1‐HIF1ɑ‐Hand1 signal pathway for CM proliferation and, in turn, for heart regeneration.

### 2.6 Cardiomyocytes‐Targeted Delivery of Hand1 Promoted Glycolytic Metabolic Transition for Raising Cell Proliferation and Improving Heart Regeneration and Cardiac Dysfunction Recovery in post‐MI Animals with Foxp1 Gain‐Of‐Function in Cardiomyocytes

So far, we demonstrated i) the Foxp1‐HIF1ɑ‐Hand1 signaling pathway in CMs is important for the metabolic transition to glycolysis, cell proliferation, and heart regeneration, and ii) deletion of Foxp1 in CMs increased HIF1ɑ‐Hand1 expression, enhancing metabolic transition, and iii) induced expression of Foxp1 in CMs inhibits cell proliferation and heart regeneration, leading to cardiac dysfunction in post‐MI hearts. In order to evaluate the potential therapeutic value of Hand1 for cardiac regeneration, we developed a cTnT promoter‐driven AAV9 vector for Hand1 targeted delivery to CMs, in order to achieve induced expression (Figure S7A–D, Supporting Information). This induced expression of Hand1 in CMs significantly reversed reduced cell proliferation in cultured Foxp1 gain‐of‐function CMs (Figure S7E–G, Supporting Information). Furthermore, we confirmed that Hand1 overexpression induced by AAV9 in human iPSC‐CMs also promotes cell proliferation (Figure S7H,I, Supporting Information).

**Figure 7:**
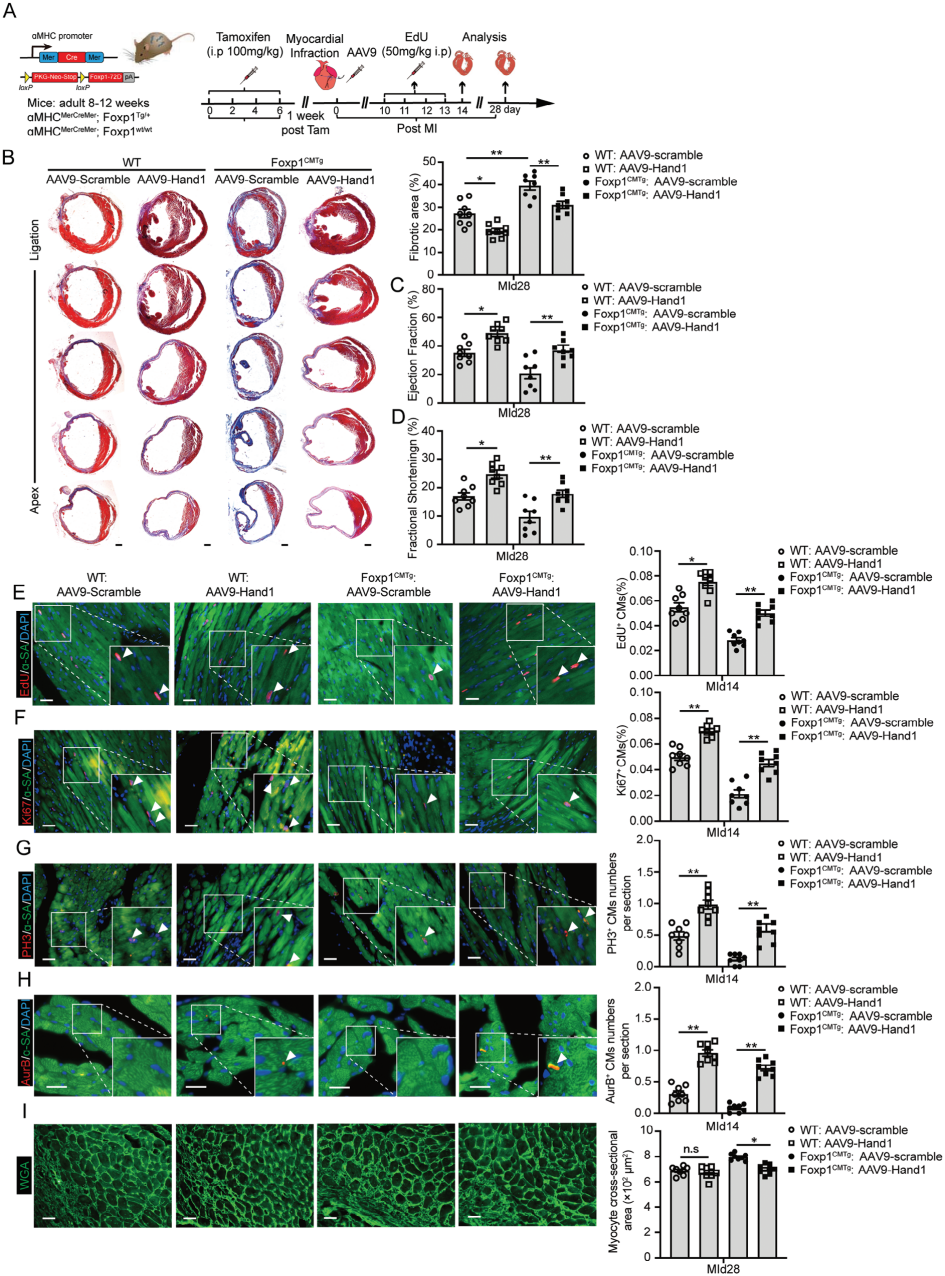
Cardiomyocyte‐targeted delivery of Hand1 promoted glycolytic metabolic transition for raising cell proliferation and improving heart regeneration and cardiac dysfunction recovery in post‐MI animals with Foxp1 gain‐of‐function in cardiomyocytes. A) Schematic diagram of intracardial injection of Hand1‐AAV9 driven by cTnT‐promoter in adult MI mice. B) Masson trichrome staining of fibrotic scar size in MId28 hearts of Foxp1^CMTg^ mice and wild‐type littermates treated by Hand1‐AAV9 or scramble‐AAV9, with representative images on the left and quantification on the right (*n =* 8). C,D) Echocardiography parameter left ventricle ejection fraction C) and fractional shortening D) evaluation of cardiac function at MId28 hearts of Foxp1^CMTg^ mice and wild‐type littermates treated by Hand1‐ or scramble‐AAV9 (*n =* 8). E–I) CM proliferation quantified by EdU incorporation E), immunostaining of Ki67 F), PH3 G), and Aurora B H) of ɑ‐SA^+^ CMs and WGA staining of CM cross‐sectional size I) at MI border zone hearts from Foxp1^CMTg^ mice and wild‐type littermates treated by Hand1‐ or scramble‐AAV9 (*n =* 8). Data are means± SEM. *p < 0.05; ** *p* < 0.01, n.s. indicates not significant. Scale bar: (B), 2 mm; (E) through (I), 50 µm.

We further examined Hand1 expression in the hearts and confirmed significant upregulation in isolated CMs at the MI border zone compared with CMs at remote zones or CMs from sham‐operated left ventricles (Figure 4B). In addition, increased Hand1 expression in ventricle CMs of Foxp1^CMKO^ mice was observed (Figure 4C). To further evaluate the in vivo heart regeneration improvement of CM‐targeted delivery of Hand1 in adult hearts, we injected cTnT‐AAV9‐Hand1 into peri‐infarct myocardial areas of post‐MI Foxp1^CMTg^ mice and wild‐type littermates. High efficacy of induced Hand1 expression in CMs was observed in mice at 14 days after injection (**Figure** **7**A; Figure S8A–C, Supporting Information). Overexpression of Hand1 in CMs ameliorates the fibrotic scar size (Figure 7B) and improves cardiac function (Figure 7C,D; Table S2, Supporting Information) at MId28 to a higher degree in Foxp1^CMTg^ mutants than in wild‐type hearts. Finally, overexpression of Hand1 in CMs had improved the MI border zone CMs proliferation (Figure 7E–H) and ameliorated CMs hypertrophy (Figure 7I) to a higher degree in Foxp1^CMTg^ mutants than in wild‐type hearts. These results demonstrated the importance of the Foxp‐HIF1ɑ‐Hand1 signaling pathway in the regulation of metabolic transition in CM proliferation and heart regeneration. Therapeutic targeting of this pathway may open new possibilities for the treatment of heart failure associated with insufficient heart regeneration following injury.

## 3 Discussion

In the current study, we analyzed the tissue and single‐cell transcriptome datasets of murine regenerative hearts^[^ ^14^ ^]^ and found a significant reduction of Foxp1 expression in the highly proliferative capacity CMs at the MI border zone. We then used both loss‐of‐function and gain‐of‐function mouse models to validate the beneficial effects of CM‐specific Foxp1 on metabolic reprogramming from FA oxidation to glycolysis, thereby enhancing cell proliferation and heart regeneration. Furthermore, we identified the HIF1α‐Hand1 transcription network as a Foxp1 direct downstream target in regulating CM proliferation. Collectively, our data provide a novel strategy to provoke post‐injury cardiac regeneration and repair (Figure S9, Supporting Information).

The mammalian neonatal heart possesses a transient capacity for cardiac regeneration during the first week after birth.^[^ ^4^, ^5^, ^6^, ^7^ ^]^ Over the past decade, various strategies have been employed to enhance postnatal heart regeneration by manipulating growth factor signaling, transcription factors, cell cycle regulatory molecules, microRNAs, and inflammatory cytokines.^[^ ^25^ ^]^ More recently, it has become evident that the energy metabolism of CMs governs developmental changes and controls the endogenous cardiac regenerative capacity.^[^ ^22^ ^]^ We demonstrated that Foxp1 deletion in CMs enhanced cardiac regenerative capacity in both neonatal AR and adult MI models. We also identified Usp20, a deubiquitinase that prevents HIF1α degradation, as a Foxp1 direct target gene. The CM‐specific deletion of Foxp1 elevates HIF1ɑ expression by inhibiting its degradation. Since HIF1α is the primary regulator of cellular energy metabolism under hypoxic conditions, its regulation by Foxp1 suggests a possible metabolic control mechanism of CM proliferation during heart generation.

The endogenous cardiac regenerative capacity in vertebrates has been reported to be under metabolic control. Metabolic reprogramming of adult CMs from glycolysis to oxidative phosphorylation is sufficient to promote cell cycle arrest of immature CMs.^[^ ^26^ ^]^ Conversely, hypoxia and metabolic reprogramming of adult CMs to a glycolytic state could facilitate cell cycle reentry, stimulate CM proliferation for heart regeneration, and improve post‐injury recovery.^[^ ^18^, ^19^, ^27^ ^]^ As described earlier, Foxp1 regulates its downstream gene HIF1α, and deletion of Foxp1 significantly elevates HIF1α expression in CMs. This elevation might mimic hypoxia and promote CM proliferation by reprogramming postnatal CMs to a glycolytic state. Moreover, CM‐specific deletion of HIF1α reversed the Foxp1 deletion‐mediated increase in CM proliferation in the neonatal AR model. These data confirm the importance of the Foxp1‐HIF1α axis in CMs during postnatal heart regeneration and improving recovery from cardiac dysfunction.

Inducing Hif‐1 activity in adult mice after MI leads to a robust regenerative response via the induction of proliferation of existing CMs supporting a link between CM metabolism and cell cycle activity.^[^ ^19^ ^]^ The transcription factor Hand1 was reported as a hypoxia‐dependent expression gene involved in the promotion of fetal CM glycolysis and in the inhibition of a considerable number of genes involved in the lipid metabolism of postnatal CMs.^[^ ^24^, ^28^ ^]^ Our study demonstrated the regulation of Foxp1 in the metabolic transition between glycolysis and FA lipid metabolism dependent via Hand1 expression. Finally, CM‐targeted delivery of Hand1 using a cTnT‐promoter‐driven AAV9 to induce the Hand1 expression in CMs of Foxp1^CMTg^ mice triggered a significant attenuation of Foxp1 gain‐of‐function‐mediated impaired CM proliferation and heart regeneration.

Our data demonstrate that the Foxp1‐HIF1α‐Hand1 signal cascade plays a crucial role in CMs for metabolic transition, cell proliferation, and heart regeneration. This suggests that Hand1 could serve as a novel molecular target for gene therapy in the treatment of heart failure. Several recent studies have indicated that long‐term induction of cardiomyocyte proliferation may have detrimental effects.^[^ ^29^, ^30^ ^]^ We found that postnatal deletion of Foxp1 in CMs did not significantly affect the morphology or function of neonatal hearts but enhanced cardiac regeneration after apical resection injury. In adult mice, we assessed cardiac recovery up to 8 weeks after MI and observed improved survival, cardiac morphology, and function in mice with CM‐specific Foxp1 deletion. Similarly, induction of Hand1 expression via AAV9‐Hand1 up to 4 weeks post‐injury resulted in improved cardiac morphology and function. Importantly, we did not observe any adverse side effects such as cardiac dysfunction, sudden arrhythmic death, or cardiomyocyte overgrowth and hypertrophy. These results highlight the therapeutic potential of modulating the Foxp1‐HIF1α‐Hand1 pathway for cardiac regeneration. However, gene therapy approaches aimed at stimulating endogenous cardiomyocyte proliferation must address concerns such as tumorigenic risks and dedifferentiation of cardiomyocytes, which can lead to aberrant gene activation and impaired contractile function. Therefore, striking a balance between regeneration and preservation of cardiac function is critical in developing effective strategies for cardiac repair, and tight control over the dosage and duration of these therapies is essential.^[^ ^29^ ^]^

Recent advancements in gene therapy technologies provide promising avenues for safe and efficient cardiac regeneration. For instance, Magadum et al.,^[^ ^31^ ^]^ developed a cardiomyocyte‐specific modified RNA (modRNA) expression system to induce Pkm2 gene expression for cardiac regeneration and repair. This technology is gaining attention due to its high efficiency, transient nature (lasting 8∼12 days), dose‐dependent, and controlled gene delivery. Additionally, Sun et al. applied this modRNA technique for cardiac regeneration in pig MI models.^[^ ^32^ ^]^ Our study contributes to this growing field by identifying Foxp1 and Hand1 as potential molecular targets for post‐injury cardiac regeneration strategies. Combining the modulation of these targets with new techniques could enhance the efficacy and safety of gene therapies aimed at promoting cardiac repair.

## 4 Conclusions

In conclusion, our study identifies the transcription factor Foxp1, a key regulator of embryonic cardiovascular development, as a critical endogenous factor governing post‐injury cardiomyocyte proliferation. Mechanistically, we found that cardiomyocyte‐specific loss of Foxp1 promotes the USP20‐HIF1α‐Hand1 signaling pathway, promoting a metabolic shift from fatty acid oxidation to glycolysis and ultimately enhancing cardiomyocyte proliferation and heart regeneration. Importantly, our findings suggest that Hand1, a downstream target in this pathway, could serve as a promising molecular target for gene therapy in the treatment of heart failure. Overall, these results may guide the development of novel molecular strategies that promote heart regeneration and repair for therapeutic intervention in heart failure.

## 5 Experimental Section

### Animals

All animal procedures were performed in accordance with the Institutional Animal Care and Use of Laboratory Animals approved by the Tongji University Institutional Animal Care and Use Committee. The conditional Foxp1 loss‐of‐function (Foxp1^flox/flox^, a kind gift from Prof. Edward E. Morrisey)^[^ ^10^ ^]^ and gain‐of‐function (Foxp1^Tg/+^)^[^ ^33^ ^]^ mice were crossed with tamoxifen‐inducible ɑ‐MHC promoter‐driven Cre line^[^ ^34^ ^]^ (ɑ‐MHC^MerCreMer^, #005657, Jackson Laboratory) to generate CM‐specific Foxp1 loss‐ and gain‐of‐function mice (Foxp1^CMKO^ and Foxp1^CMTg^, respectively). Similarly, HIF1ɑ conditional loss‐of‐function mice (HIF1ɑ^flox/flox^, NM‐CKO‐190065, Shanghai Model Organisms Center), were crossed with Foxp1^CMKO^ mice to generate cardiomyocytes Foxp1 and HIF1ɑ double knockout mice. All strains were crossed on a C57BL/6J background (purchased from Shanghai Slac Laboratory Animal Co. Ltd). Neonatal apical resection (AR) and adult myocardial infarction (MI) mouse models were performed with detailed descriptions in the Experimental Section of Supporting Information. Details of adeno‐associated virus 9 (AAV9) injection for CM‐specific Hand1 overexpression in vivo to rescue Foxp1^CMTg^ mediated impaired cardiac regeneration are provided in Supporting Information.

### Datamining Analysis, Molecular Methods, and Reagents

The details of data mining analysis of neonatal heart regeneration database, RT‐qPCR, immunoblotting, hematoxylin and eosin (H&E) staining, Masson's trichrome staining, Triphenyl tetrazolium chloride (TTC) staining and immunostaining, expression vectors, cell culture/transfection and proliferation/migration assay, in vitro siRNA transfection, ChIP assay, luciferase reporter assay, non‐targeted metabolomic study were provided in Supporting Information.

### Statistical Analysis

Statistical analysis was performed using SPSS software version 25.0 and GraphPad Prism 9. Kaplan‐Meier survival curves were used to examine mouse survival rates, and the differences were analyzed with the log‐rank (Mantel‐Cox) test. Parametric data are presented as means ± S.E.M. for at least three independent assays unless otherwise noted. All data passed the normality and equal variance before analysis. Student's t‐test was used for two‐sample comparisons, one‐way ANOVA with Tukey post‐hoc tests for comparisons between multiple groups, and two‐way ANOVA for comparisons between multiple groups when there were two experimental factors. *p*‐Values of <0.05 were considered as statistical significance.

### Ethical Statement

All animal procedures were performed in accordance with the Institutional Animal Care and Use of Laboratory Animals approved by the Tongji University Institutional Animal Care and Use Committee. The Institutional Review Board (IRB) of this study was obtained from Shanghai East Hospital and Tongji University (TJBB00222101).

## Conflict of Interest

The authors declare no conflict of interest.

## Author Contributions

Y. F. W., X. Y. W., J. F., and X. L. C. contributed equally to this work. J. L. and Y. Z. Z. conceived and designed the study; Y. F. W., X. Y. W., J. F., X. L. C., T. Z., S. P., W. Z. B., W. R. W., and Y. S. L. performed experiments and acquired data; T. X. and W. R. W. analyzed the single‐cell database; H. K. W. provided the Foxp1 gain‐of‐function mice; B. T., P. C., sp S. G. Z., Q. Z., and L. Z. gave suggestions and revised the manuscript; Z. M. L. and J. J. P. contributed grant support and gave suggestions for study design and manuscript revision. All authors contributed to drafting and revising the article and approved the submitted version.

## Supporting information



Supporting Information

## Data Availability

The data, methods used in the analysis, and materials used to conduct the research are available from the corresponding author upon reasonable request.
