# Supplementary material for: Cardiomyocyte Foxp1‐Specific Deletion Promotes Post‐injury Heart Regeneration via Targeting Usp20‐HIF1ɑ‐Hand1 Signaling Pathway
Source: Adv Sci (Weinh). 2025 Feb 3;12(12):2412124. doi: 10.1002/advs.202412124 (PMC11948019; doi:10.1002/advs.202412124)
Supplement: Supplementary file 1 — Supporting Information [file ADVS-12-2412124-s001.pdf]

ADVANCED SCIENCE
Open Access

Supporting Information

for *Adv. Sci.*, DOI 10.1002/advs.202412124

Cardiomyocyte Foxp1-Specific Deletion Promotes Post-injury Heart Regeneration via Targeting Usp20-HIF1α-Hand1 Signaling Pathway

Yanfang Wang, Xiaoyu Wang, Ji Fang, Xiaoli Chen, Teng Xu, Tao Zhuang, Sheng Peng, Wenzhen Bao, Wenrun Wu, Yushi Lu, Haikun Wang, Brian Tomlinson, Paul Chan, Shougang Zhuang, Qi Zhang, Lin Zhang, Zhongmin Liu, Jingjiang Pi, Yuzhen Zhang* and Jie Liu**

Supporting Information

Title: Cardiomyocyte Foxp1-Specific Deletion Promotes Post-injury Heart Regeneration via Targeting Usp20-Hif1α-Hand1 Signaling Pathway

*Author(s), and Corresponding Author(s)**

Yanfang Wang, Xiaoyu Wang, Ji Fang, Xiaoli Chen, Teng Xu, Tao Zhuang, Sheng Peng, Wenzhen Bao, Wenrun Wu, Yushi Lu, Haikun Wang, Brian Tomlinson, Paul Chan, Shougang Zhuang, Qi Zhang, Lin Zhang, Zhongmin Liu, Jingjiang Pi*, Yuzhen Zhang*, Jie Liu*

Supporting Experimental Section

Datamining analysis of single nucleus RNA-sequencing neonatal heart regeneration database

Single nucleus RNA sequencing (snRNA-seq) dataset for cardiomyocytes were obtained from the Gene Expression Omnibus (GEO) database under accessions GSE130699[1]. In brief, data from 8 samples were processed using the Seurat package (version 5.1.0). All genes expressed in < 3 cells were removed, the number of genes expressed per cell > 300 as low, and < 7000 as high cutoff. Additionally, cells with mitochondrial DNA-derived gene expression exceeding 10% were removed to ensure data quality. To visualized the data, dimensionality reduction was performed using Seurat, and cells were projected into 2D space using Uniform Manifold Approximation and Projection (UMAP). Gene expression values were normalized using the "LogNormalize" method in Seurat's normalization function. Principal component analysis (PCA) was then applied to the normalized data for initial feature selection and dimensionality reduction. For clustering, we utilized the weighted Shared Nearest Neighbor (SNN) graph-based clustering method. Cardiomyocytes were identified based on the expression of the Myh6 gene. Following this, cardiomyocytes were further divided into five clusters according to the classification scheme described by Cui et al. Specifically, the regenerative cardiomyocyte population in cluster 4 (CM4) was identified by the expression of markers Atp5b, Mdh2, Sod2, and Mb, while the remaining clusters were classified as non-regenerative cardiomyocytes.

Tamoxifen induction of cre recombinase for cell specific gene deletion and overexpression

Tamoxifen (T5648, sigma) was dissolved in 90% peanut oil / 10% alcohol and intragastrically injected (40 µg per day) at postnatal day 0 to 3 in neonatal mouse, and intraperitoneally injected (100 mg/kg per dose) every other day for a total of 4 injection in adult mice for induction of gene deletion or overexpression.

Neonatal mouse apical resection model

The neonatal mouse apical resection model for cardiac regeneration were performed as previously described.[2, 3] Briefly, neonatal mouse of postnatal day 3 were anesthetized by hypothermia on ice for ~3-5 min. Lateral thoracotomy at the fourth intercostal space was performed by blunt dissection of the intercostal muscles following skin incision. Then, iridectomy scissors were used to resect the apex of P3 hearts until the left ventricular chamber was exposed. Following apical resection, neonates were removed from the ice bed, thoracic wall incisions were sutured with 7-0 suture, and the skin wound closed with 6-0 suture. Neonates were placed under a heat lamp and warmed until recovery and then placed into feeding cage.

Adult mouse myocardial infarction model

The adult mouse myocardial infarction (MI) model was performed as previously described.[4] In brief, 8-week-old Foxp1^{CMKO} mice or Foxp1^{CMTg} mice and wild-type littermates were anesthetized with 1% isoflurane in a chamber and mechanically ventilated (isoflurane 1% vol/vol) with a rodent respirator device. The mice were placed in a left supine position on a heating pad (37 °C), and the heart was exposed via thoracotomy at the fourth left intercostal space. The pericardium was then opened, and the left coronary artery was permanently ligated with an 8-0 suture. Ligation was considered successful when the left ventricle became pale. The chest was then closed with 6-0 suture and mouse put to feeding cage after recovery.

Non-targeted metabolomics procedure and analysis

Age matched P3ARd7 or sham operated neonatal heart and Foxp1^{CMKO}, Hif1α^{CMKO} or Foxp1; Hif1α in CMs double deletion mouse hearts were sent to Applied Protein Technology Co., Ltd. (Shang, China) for metabolite extraction and non-targeted metabolomics analysis. For metabolite extraction, the samples were frozen in liquid nitrogen immediately after dissection, the hearts were cut on dry ice and 200 µl H_2O and 5 ceramic beads were homogenized, then 800 µl methanol/acetonitrile (1:1, v/v) were added to homogenized solution and the mixture was centrifuged for 15 min (14,000 g, 4°C). The supernatant was dried in a vacuum centrifuge and the samples were re-dissolved in 100 µl acetonitrile/water (1:1, v/v) solvent.

The LC-MS/MS analysis were performed by an UHPLC system (1290 Infinity LC, Agilent Technologies) coupled to a quadrupole time-of-flight (AB Sciex TripleTOF 6600). For HILIC separation, samples were analyzed using a ACQUIY UPLC BEH column (1.7 µm 2.1 × 100 mm, Waters). The mobile phase contained 25 mM NH4OAc and 25 mM NH4OH in water (A) and acetonitrile (B). In both ESI positive and negative modes, the gradient was 85% B for 1 min and was linearly reduced to 65% in 11 min, and then was reduced to 40% in 0.1 min and kept for 4 min, and then increased to 85% in 0.1 min, with a 5 min re-equilibration period employed. For RPLC separation, a ACQUIY UPLC HSS T3 column (1.8 µm 2.1 × 100 mm, waters) was used. In ESI positive mode, the mobile phase contained 0.1% formic acid (A) and 0.1 acetonitrile with 0.1% formic acid (B); and in ESI negative mode, the mobile phase contained 0.5 mM ammonium fluoride in water (A) and acetonitrile (B). The gradient was 1% B for 1.5 min and was linearly increased to 99% in 11.5 min and kept for 3.5 min. Then it was reduced to 1% in 0.1 min and a 3.4 min of re-equilibration period was employed. The gradients were at a flow rate of 0.3 mL/min, and the column temperatures were kept constant at 25°C. A 2 µL aliquot of each sample was injected. The ESI source conditions were set as follows: Ion Source Gas1 (Gas1) as 60, Ion Source Gas2 (Gas2) as 60, curtain gas (CUR) as 30, source temperature: 600 °C, IonSpray Voltage Floating (ISVF) 5500 V. In MS only acquisition, the instrument was set to acquire over the m/z range 60-1000 Da, and the accumulation time for TOF MS scan was set at 0.20 s/spectra. In auto MS/MS acquisition, the instrument was set to acquire over the m/ z range 25 1000 Da, and the accumulation time for product ion scan was set at 0.05 s/spectra. The product ion scan is acquired using information dependent acquisition (IDA) with high sensitivity mode selected. The parameters were set as follows: the collis ion energy (CE) was fixed at 35 V with 15 eV; declustering potential (DP), 60 V (+) and 60 V (−); exclude isotopes within 4 Da, candidate ions to monitor per cycle: 10.

MS raw data files from metabolomics were converted to the MzXML files using ProteoWizard, and processed by R package XCMS. For peak picking, the following parameters were used: centWave m/z = 25 ppm, peakwidth = c (10, 60), prefilter = c (10, 100). For peak grouping, bw = 5, mzwid = 0.025, minfrac =0.5 were used. CAMERA (Collection of Algorithms of MEtabolite pRofile Annotation) was sued for annotation of isotopes and adducts. In the extracted ion features, only the variables having more than 50% of the nonzero measurement values in at least one group were kept. Compound identification of metabolites was performed by comparing of accuracy m/z value (<25 ppm), and MS/MS spectra with an in-house database established with available authentic standards. After normalized to total peak intensity, the processed data were analyzed by R package (ropls), where it was subjected to

multivariate data analysis, including Pareto-scaled principal component analysis (PCA) and orthogonal partial least-squares discriminant analysis (OPLS-DA). The 7-fold cross-validation and response permutation testing was used to evaluate the robustness of the model. The variable importance in the projection (VIP) value of each variable in the OPLS-DA model was calculated to indicate its contribution to the classification. Metabolites with the VIP value >1 was further applied to Student's t-test at univariate level to measure the significance of each metabolite, the p values less than 0.05 were considered as statistically significant.

AAV vectors construction and adult mouse intracardiac injection

Hand1 overexpressing adeno-associated virus driven by cardiac specific gene cTnT promoter (AAV9-cTnTp-mHand1-EGFP) or control viral vectors (AAV9-cTnTp-EGFP) were constructed by Hanbio Biotechnology Co., Ltd. and was intracardially injected to the peri-infarction site of adult mice with a 30-gauge insulin syringe at a dose of 4×10^{11} viral genome particles per animal just after MI operation as previously described.[5] The hearts were collected at 14 days after AAV injection to measure Hand1 overexpression in CMs.

The cloned sequence for mouse Hand1 ：

5‘ ATGAACCTCGTGGGCAGCTACGCACATCATCACCATCATCACCACTCACACCC
GCCGCACCCCATGCTCCACGAACCCTTCCTGTTTGGCCCGGCCTCGCGTTGCCAC
CAGGAGCGGCCTTACTTCCAGAGCTGGCTGCTGAGCCCGGCTGATGCTGCCCCA
GATTTCCCTGCCGGCGGGCCACCACCTACCACCGCAGTAGCAGCGGCTGCCTAT
GGTCCCGATGCCAGGCCGAGTCAGAGCCCAGGTCGGCTGGAGGCTCTTGGAAGC
CGCCTGCCCAAACGAAAAGGCTCAGGACCCAAGAAGGAGAGGAGACGCACAGA
GAGCATTAACAGCGCGTTCGCGGAGCTGCGTGAGTGCATCCCCAATGTGCCCGC
CGACACCAAGCTCTCCAAGATCAAGACTCTGCGCCTGGCTACCAGTTACATCGC
CTACTTGATGGACGTGCTGGCCAAGGATGCACAAGCAGGTGACCCCGAGGCCTT
CAAGGCTGAACTCAAAAAGACGGATGGTGGTCGCGAAAGCAAGCGGAAAAGGG
AGTTGCCTCAGCAGCCCGAAAGCTTCCCTCCTGCCTCGGGGCCCGGCGAGAAGA
GGATTAAAGGGCGCACCGGCTGGCCTCAGCAAGTCTGGGCGCTGGAGCTAAACC
AGTGA 3’

Echocardiography Analysis

Echocardiography was performed to evaluate the cardiac geometry, and systolic function as previously described.[6] The Visual Sonics high-resolution Vevo2100™ ultrasound system (VisualSonics Inc., Canada) with a 30-MHz linear array ultrasound transducer (MS-400, VisualSonics Inc.) was used. In brief, mice were anesthetized with 2.0% isoflurane until the heart rate stabilized at 400 to 500 beats per minute. Parasternal long-axis images were acquired in B-mode with appropriate position of the scan head to identify the maximum LV length. In

this view, the M-mode cursor was positioned perpendicular to the maximum LV dimension in end-diastole and systole, and M-mode images were obtained for measuring wall thickness and chamber dimensions. LV ejection fraction (LVEF) and fractional shortening (LVFS) were calculated automatically.

Histology

Mice were perfused with cold PBS and hearts were hearts harvested, fixed with 4% paraformaldehyde for 24 hours, and embedded in paraffin wax or OCT. Serial sections were obtained at 6 μm intervals for paraffin embedded tissue and 8 μm intervals for OCT embedded tissue. Serial sections were stained with Masson's trichrome for detection of cardiac fibrosis and Alexa Fluor™ 488 conjugated wheat germ agglutinin (WGA, W11261, Invitrogen) for measurement of cardiomyocyte size in vivo by myocyte cross-section areas.[7] Images were captured by a Leica microscope (DM6000B, Leica, Germany).

For the neonatal regeneration model, the fibrotic scar area was quantified by calculating the mean fibrotic area from the largest two ventricular longitudinal section by Image J software based on Masson's trichrome staining. While for the adult myocardial infarction model, the fibrotic scar area was quantified by examining 5 serial sections from the apex to the ligation site and calculating the average percent fibrotic area of the total ventricle area by Masson's trichrome staining. Similar methods were used to evaluate cardiomyocyte hypertrophy by calculation of the average border zone cardiomyocyte cross-section area in WGA staining heart sections, relative capillary density as average capillaries per cardiomyocytes by calculation of the isolectin B4 positive capillary numbers divided by WGA staining cardiomyocyte numbers in IB4/WGA co-staining cardiac sections,[8] and arteriole density was also quantified in α-SMA staining sections.

Immunostaining was performed using the following antibodies: rabbit anti-α-SMA (2.5 μg/ml, 14395-1-AP, Proteintech), rabbit anti-Foxp1 (2 μg/ml, gift from Prof. Edward E Morrisey[9]), mouse anti-α-sarcomere actinin (2.5 μg/ml, ab9465, Abcam), rabbit anti-α-sarcomere actinin (1.5μg/ml, ab68167, Abcam) rabbit anti-PH3 (2 μg/ml, ab80612, Abcam), rabbit anti-Ki67 (5 μg/ml, ab15580, Abcam), mouse anti-aurora B (20 μg/ml, ab3609, Abcam), mouse anti-PCM1 (2 μg/ml, ab16637, Abclonal), and FITC or Dylight 594-conjugated Isolectin B4 (5 μg/ml, FL-1201/DL1207, Vector Laboratories). After washing with PBST, the cells were incubated with Alexa Fluor 488-conjugated donkey anti-mouse secondary antibodies (2 μg/ml, A21206, Invitrogen) and Alexa Fluor 568- conjugated donkey anti-rabbit secondary antibodies

(2 μg/ml, A10042, Invitrogen) for 45 min. Slides were mounted with Vectashield mounting medium containing DAPI (Vector Laboratories, Burlingame, USA).

Evans blue perfusion and 2,3,5-triphenyltetrazolium chloride (TTC) staining

To distinguish among infarcted area, area at risk, and unaffected remote myocardium after adult myocardial infarction (MI) injury, a water-soluble dye (Evans blue) was injected retrogradely via the descending thoracic aorta to perfuse the coronary vasculature and myocardium, then followed by TTC staining. The percentage of infarcted (white), area at risk (red), and unaffected remote (blue) myocardium 1 day after MI was quantified by using Image J software as described.[10]

EdU incorporation assay for assessment of cardiomyocyte proliferation

For NMCMs, the medium was replaced with medium containing 10 μM EdU (c10338, Thermo Fisher Scientific) 48 h after transfection, and cells were fixed 12 h later. For EdU assay of neonatal heart section, a single injection of 500 μg EdU per animal was induced intraperitoneally a 6.5 day post apical resection. For adult mice cardiomyocytes EdU assay, a serial injection of 1000 μg EdU per animal / day was induced intraperitoneally from 10-13 days post MI. For EdU staining, the rehydrated heart sections and cells were incubated with Click-iT EdU 555 Imaging Kit reagents (c10338, Thermo Fisher Scientific) to reveal EdU incorporation according to the manufacturer's instructions.

Primary culture of neonatal cardiomyocytes and isolation of adult ventricular cardiomyocytes

Cardiomyocytes (CMs) were isolated from neonatal mice (NM) at age of postnatal P1-P3d for NMCMs primary culture. Briefly, the mouse ventricles were separated and cut into pieces, then dissociated in calcium-free HBSS (Gibco) containing 0.125 mg/mL trypsin (Gibco), 0.1mg/mL collagenase type IV (sigma) and 10 mg/mL DNase II (Sigma). Digestion was performed at 37°C by continually stirring, and the supernatant was collected every 5 min in a tube to terminate digestion with HBSS containing 10% FBS. The digestion procedure was repeated ~ 8-10 times. When the tissue was completely digested, all collected supernatant was centrifuged at 1000 rpm for 10 min and resuspended in DMEM (Gibco) supplemented with 10% FBS and 100 mM 5-bromo-20-deoxyuridine (B5002, Sigma). The resuspended cells were passed through a cell strainer (100 μm, BD), seeded onto 10 cm plastic dishes for 2 h at 37°C to remove fibroblasts, and then plated on 1% gelatin-coated plastic culture dishes at an appropriate density. The cells were stained with cardiomyocyte specific cTNT and analyzed by

flow cytometry and counting of $cTNT^+$ cells, the purity of NMCMs were over 90%. The medium was changed to low-serum medium after 24 h and the cells were cultured for 48 h before use.

Isolation of adult cardiomyocytes were performed as previously reported. [11] Briefly, mice (8-10 weeks of age) were anesthetized with phenobarbital (80 mg/kg). The chest was opened to fully expose the heart. The inferior vena was cut, and the heart was immediately flushed by injection of 10 mL ethylenediaminetetraacetic acid (EDTA) buffer into the right ventricle. The ascending aorta was clamped with Reynold's forceps, the heart was transferred to a 60-mm dish containing fresh EDTA buffer. Digestion was achieved by sequential injection of 10 mL EDTA buffer, 3 mL perfusion buffer, and 30 to 50 mL collagenase buffer into the left ventricle (LV). Constituent chambers (atria, LV, and right ventricle) were then separated and gently pulled into 1-mm pieces using forceps. Cellular dissociation was completed by gentle trituration, and enzyme activity was inhibited by addition of 5 mL stop buffer. The cell suspension was passed through a 100-μm filter, and cells underwent 3 sequential rounds of gravity settling, using 3 intermediate calcium reintroduction buffers to gradually restore calcium concentration to physiological levels. The cell pellet in each round was enriched with myocytes and ultimately formed a highly pure myocyte fraction. CMs yields and the percentage of viable rod-shaped cells were quantified using a hemocytometer. CMs were then resuspended in prewarmed plating media and plated onto laminin precoated plates or coverslips.

Cell gene-siRNA transfection

For gene silencing studies, siRNAs against mouse *Foxp1*, *Hif1a* and *Hand1* were transfected into NMCMs at a final concentration of 50 nM using Lipofectamine RNAiMAX (13778, Invitrogen). All gene siRNA sequences were listed in Table S4.

Glucose utilization and lactate production assays

For measurement of glucose utilization and lactate production, isolated P1-P3d NMCMs were seeded onto a laminin coated 24-well plate (1×10^5 per well). NMCMs were transfected with *Foxp1*, *Hif1a*, *Hand1 or scramble* siRNAs for 48 hours for gene knockdown. The cells were changed with 500 μl fresh culture medium and cultured for 48 hours with the last 12 hours under hypoxia condition (5% CO_2, 1% O_2 and 94 % N_2), and supernatant harvested. Supernatant concentration of D-glucose and lactate measured D-glucose Content Assay Kit (AKSU001C, Boxbio Science & Technology) and Lactic Acid Content Assays kit (AKAC001M, Boxbio Science & Technology) respectively. Glucose utilization was calculated as the glucose concentration in fresh culture medium subtracting that in the supernatants.

Cell immunostaining

NMCMs cultured on laminin precoated coverslips in 24-well plates were fixed with 4% paraformaldehyde (PFA) in PBS, washed and permeabilized with 0.1% Triton X-100 PBS. The cells were blocked with 1% goat serum for 1 hour and incubated with primary antibodies: mouse anti-α-sarcomere actinin (2.5 µg/ml, ab9465, Abcam), rabbit anti-α-sarcomere actinin (1.5µg/ml, ab68167, Abcam), rabbit anti-PH3 (2 µg/ml, ab80612, Abcam), mouse anti-aurora B (20 µg/ml, ab3609, Abcam), overnight. After washing, the cells were incubated with Alexa Fluor 488-conjugated donkey anti-mouse secondary antibodies (2 µg/ml, A21206, Invitrogen) and Alexa Fluor 568- conjugated donkey anti-rabbit secondary antibodies (2 µg/ml, A10042, Invitrogen) for 45 min. Then, the cover slips were mounted with Vectashield mounting medium containing DAPI (Vector Laboratories, Burlingame, USA). The immunostaining images were captured under a Leica fluorescent microscope (DM6000B, Leica, Germany).

RNA purification and RT-qPCR

For quantitative PCR (qPCR), RNA from heart tissues, isolated neonatal or adult cardiomyocytes or culture cell lines was extracted by TRIzol™ (Invitrogen) and cDNA synthesized by SuperScript First Strand Synthesis System (Invitrogen). RT-qPCR was performed using PowerUp™ SYBR™ Green master mix (A25741, Applied Biosystems) on a QuantStudio™ 6 Flex Real-time PCR system (Applied Biosystems, USA) following the manufacturer's instructions. GAPDH was used as internal control. Primers used for quantitative real-time PCR were included in Table S3.

Western Blot Analysis

Tissue homogenates or Cells pellets were extracted in RIPA lysis buffer (P0013C, Beyotime) containing protease inhibitor cocktail (539131, Calbiochem). Protein concentrations were determined by a BCA kit (23225, Pierce). Equal amounts of cell extracts (30 µg total protein) were electrophoresed on sodium dodecyl sulfate-polyacrylamide gels, and then transferred onto polyvinylidene difluoride membranes (ISEQ08100, Millipore). The membranes were blocked in 5% non-fat dried milk in TBS-T at room temperature for 2 hours and incubated with indicated primary antibodies at 4°C overnight. The primary antibodies used in the study were: Rabbit anti-FOXP1 (1 µg/ml, gift from Prof. Edward E Morrisey [9]), anti-GAPDH (1 µg/ml, #5174, Cell signaling technology), mouse anti-HIF1α (1 µg/ml, ab1, Abcam), rabbit anti-USP20 (1 µg/ml, ab72225, Abcam), mouse anti-HAND1 (1 µg/ml, ab279390, Abcam), rabbit anti-CCNB1(1 µg/ml, A16038, Abclonal), rabbit anti-CCND1 (1µg/ml, A19038, Abclonal), mouse anti-HA (1 µg/ml, AE008, Abclonal), mouse anti-His (1 µg/ml,

AE003,Abclonal). The membranes were washed three times with TBS-T for 10 min, and then incubated with Goat Anti-Mouse IgG (H+L) secondary antibody (31430, Thermo Fisher Scientific) or Goat Anti-Rabbit IgG (H+L) secondary antibody (32460, Thermo Fisher Scientific) at room temperature for 1 hour. After washing in TBS-T for another three times, the signals were detected by using a chemiluminescence kit (12630, Cell Signaling Technology). Experiments were repeated three times and the target protein level was quantified by Image J and normalized to internal control.

Cleavage Under Targets and Tagmentation (CUT&Tag)

The CUT&Tag assay was conducted following the manufacturer's instructions. Briefly, 5×10^5 primary myocardial cells were incubated with concanavalin A-coated magnetic beads (ConA beads) at room temperature for 10 minutes. The cells were then sequentially incubated with a primary antibody (anti-Foxp1), a secondary antibody (Goat anti-Rabbit IgG H&L, Ab207-01-AA, Vazyme), and Hyperactive™ pA/G-Tnp Pro Transposase, and then fragmented. The fragmented DNA was extracted from the samples and amplified via PCR. CUT&Tag libraries were constructed using Hyperactive™ In-Situ ChIP Library Prep Kit (TD901, Vazyme) and sequenced on an Illumina Hiseq 2000/2500 platform. Quality control and preprocessing of the sequencing data were performed using Fastx (version 0.20.0) and Bowtie (version 0.12.8) to remove low-quality reads and sequencing adapters before genome mapping. Finally, genes of interest were visualized using Integrative Genomics Viewer (IGV, version 2.16.2).

Chromatin immunoprecipitation (ChIP) assay

Chromatin DNA was extracted from NMCMs to investigate the association of Foxp1 with the USP20 and Hif1α with the Hand1 gene. We utilized a commercially available ChIP kit (17-295, Millipore) following the manufacturer's instructions, with the exception of using protein-G agarose beads to preclear the chromatin. The isolated NMCMs were cross-linked in 1% formaldehyde to stabilize protein-DNA interactions. Following cross-linking, the chromatin was immunoprecipitated using either rabbit anti-Foxp1, mouse anti-Hif1α antibodies, or rabbit / mouse IgG antibodies as negative controls. After immunoprecipitation, the cross-links were reversed, and the purified DNA was subjected to PCR amplification using primers listed in Table S5.

Luciferase Reporter Assay

For the luciferase assay of USP20, the gene promoter sequences of USP20, from -0 to -2kb containing 3 foxp1 binding sites were inserted into a pGL4.10 promoter luciferase vector. The cloned sequence was:

5‘ CTCTGCTTCAGGCCCTGCCATGATTCCCCCAATAACTGAGGATGATCAGGAAG
GGTACAAGTTGTTTTGGTCCTGGTCACAGCAGTAGGAAGCAAACTGAGACAATTG
GGATTACATAACTGAGAGAGTAGTGGGCAGTAGTTACAAAAGCCAAAGAAGAAC
CCAGTGCTCTGCTCGCTGTGAATGGTGAAATGGGGGGTCGGGTGAGAGGCAATG
AAGCAGTTGGCGCACGCTCCTGCAAAGCCATTTGATATTCTGGAAATCACAGAGT
AAGGTTCTGCCAGTCAAGGGATCTGTATCAGAAGACATATGCCAGAGGAAAATTT
ATCAAAATGACCCTAAAACCTGAAGCCAAGGCTTGACAGGGACTGAGTAAGCAC
ACCAGCACTGAGCTATCCCCACCATTTCTTGTTTACTTTATGACCCAGGTCTCATA
GTTGCTCAGTCAGGCCTTAAACCTGTGATCTTCCCACACAGCTGGGACTACAGTG
CTATGGCACTAGGATGGGCCTTGTTGATGCTGAGACCAGTTTCACTCTGCAGGCC
CGGCTGGCACCAAACTCATGGTAATCCCACTGTTTCAGTTTCTCAAGTCTGGGAG
TCTAGGTGTGAGGCAGCACACAATCCCAGTCAATCTGGTAGCAAGTTATAGTGCA
CACCCCTGGCAATGCACACGACGCAGCCTTCTAAAAAGGTTTTCTGGAAAACACA
CACCATGGTCAGAGGCTCACAGTAATATGATGATTTAACTCTTTAAAAGCATGGG
ATAAACTAAAAAAGAAATAGGTGGGAAATAAATGGAAATATTATTATAAATGCT
GTCTCTCTACCTCAAGTTTTTTTCTTTTTCTTTATTCTTCTTATGAAGCCTAGGTTG
CCCTCAAACTCCCTGTGATAGGATTGCACCAAACCAGGCTATTGTTTATTTATTAT
ACTTTTTAACCTAAGTTTCCCAAATTCTTATCTCTTAACACGTTGTCAATCACAAT
TCATCAGTCCAGCTAAAATTTACAGTTAGTGACTTGGAGCAGACCCATAGACCAT
CAGGTTCAGGGCATGTGACAAACATTTATAAAGAACCTTAGTTCCCTCTATGATC
AGTAGAACTGTATGTCAAAGGAGGGATTTGTGACTGCGACCCTAGCTGGAAATTT
TTGTTTTAGCTGGAAATCAATGACGCCGCGACCACCCCCGTGGTACCCACCTCAC
CCCATTTGGCCTCTTCCGCAAGTTCTGCACCTCTCGGGTTTCTTCCAGTTTTAGCCT
GGGGGAAAAAAAGGAATAAAAAGTGACGCACAGGTTGAGACCTGCGGAACTCA
AAAGCCAAACTCCGTTCTAAGGACATGTCCCTTTGCCGAGTCCCTGATCCAGTGG
TCATCGGCAGTCAGACCCATGCGACCCCGGGGCACCTCTCCCAGCCGCGATGTGG
CCTTATCCCTAGGAGCCTCTGCGTGGCGCTGGCCGGTATCCAGTCCCAAGCCTGT
CTCAGCAACACGTACCGAACCTCCTCTGATTCTTGTTCATCCTCCTCCGACTCCGA
GTCAGCCCGGCGCCGGCGGAATGTTTTCCCAGTAATCCGCATAGCGGAACCAGAC
CCTTGGCGCTGCGTTACCAGACGCGTAGTCCAGGGAACCTCTCTCTTCCGGCGCG
CAGAAGTGTCGTCAGAGCGTCGGCTGCACTCAGACCCGCCTCCGCCTTGCCAGGC
CACACCCCAAGCCCGTAGGGCCCCGCCTCAGCAGGGCAGGCAACCACTCCGAAC
TCCGAAAAGCAAGGGCAGGTATTGCCGCGTGGCCACACCCTCGACCACGCCCATC
GAGTAACCATGCTCACCGCTCAACAGGACGGCCTTCGAACCACACGGTGAAACC
CCAGCAGCACGGCCACTCCCCCGACTCCCGGCATGCTTGGGCACTCACCACTCGC
ACGGCCACGCCTCCGGCGTGCCCGGTACCGCCCTCTAGTAGGCTGTGACCTGTGA
CACCGCCCCCGCTCCTGGCATAG 3’

For the luciferase assay of Hand1, the gene promoter sequences of Hand1, from -0.8 to -1.2 kb containing 4 HIF1α binding sites were inserted into a pGL4.10 promoter luciferase vector. The cloned sequence was:

5'AGGGCAGCTCCATTTCCAGGATCTCCTCCCCCACCCCGCCCCCCACCCCCGACAGGGTT
TCCCTGTGTAGCCCTGGCTGTCCTGGAACTCACGCTGTAGACCAGGCTGGCCTCTAACTCA
GAAATTCGCCTGCCTCTGCCTCCCAAGTGCTGGGATTAAAGGCGTGCGCTGCCACGCCCG
GCTGGATCTCTCTTCTGTAGCTAAGTAACACTCTGACCCCAGGAGCCTCCCTCCGTCCCAG
TATTCCACACAGCAGCCGCTCGGTGCTCGGTTCGTACACACGGAATCCCTCATTCACCCTT
TCTGAGTTCTCTCACTTCTTAAGAAAAAAAAAATCCATTAGTGTGTGCAGATGAGTGTGT
GCGGGCGTGTGAACGGGAGTGTGGAGGTCAAAGGGCAA 3'

Luciferase activity was determined after transfection for 48 hours using Dual-Glo^{TM} Luciferase assay system (E2920, Promega). Individual luciferase activity was normalized to the corresponding renilla-luciferase activity,[12] and the data presented are the average of three luciferase assays performed in triplicate.

Ubiquitylation Assays

For in vitro ubiquitylation assay, Hif1α-HA, ubiquitin (Ub)-His were transfected into HEK293T cells with Lipofectamine^{TM} 3000 (Thermo Fisher Scientific, L3000001) following the manufacturer's instructions. 48 hours later, the cells were treated with MG132 (50 μM) for 4 hours before being harvested. Then the cells were washed with PBS, pelleted, and lysed in cold RIPA buffer containing protease inhibitors and 20 mM N-ethylamaleimide (NEM) and directly boiled for 15 min. The lysates were diluted 10 times with NETN buffer containing protease inhibitors, 20 mM NEM, and 1 mM iodoacetamide and centrifuged to remove cell debris. The cell extracts were subjected to immunoprecipitation with the indicated antibodies at 4°C overnight and protein A/G agarose beads (Beyotime, P2055) for further 3 hrs at 4 °C. After washing with NETN buffer containing 20 mM NEM and 1 mM iodoacetamide) for four times, the immunocomplexes were separated by SDS-PAGE and blotted with mouse anti-His antibody (1 μg/ml, AE003, Abclonal).

Supporting Tables

Table S1: Echocardiology and weight analysis of neonatal heart apical resection model

Echocardiology Data	Sham WT	Sham Foxp1^{CMKO}	Sham 12w WT	Sham 12w Foxp1^{CMKO}	P3ARd21 WT	P3ARd21 Foxp1^{CMKO}	P3ARd21 Hif1α^{CMKO}	P3ARd21 Foxp1;Hif1α^{DKO}	P3ARd21 Foxp1^{CMTg}
IVS; d (mm)	0.509 ± 0.021	0.698 ± 0.174	0.792 ± 0.034	0.718 ± 0.032	0.553 ± 0.065	0.578 ± 0.074	0.646 ± 0.069	0.69 ± 0.05	0.546 ± 0.064
IVS; s (mm)	0.819 ± 0.063	0.964 ± 0.200	1.184 ± 0.045	1.072 ± 0.05	0.820 ± 0.067	0.973 ± 0.097	0.896 ± 0.073	1.092 ± 0.112	0.875 ± 0.114
LVID; d (mm)	3.123 ± 0.092	2.898 ± 0.217	3.575 ± 0.053	3.476 ± 0.149	3.182 ± 0.111	2.793 ± 0.141	3.387 ± 0.148	3.143 ± 0.225	3.272 ± 0.060
LVID; s (mm)	2.077 ± 0.105	1.860 ± 0.290	2.2 ± 0.097	2.185 ± 0.27	2.288 ± 0.079	1.633 ± 0.189	2.378 ± 0.123	1.718 ± 0.287	2.236 ± 0.100
LVPW; d (mm)	0.693 ± 0.051	0.754 ± 0.119	0.738 ± 0.027	0.8 ± 0.07	0.720 ±0.062	0.729 ± 0.076	0.741 ± 0.072	0.757 ± 0.114	0.753 ± 0.075
LVPW; s (mm)	0.839 ± 0.053	0.919 ± 0.131	1.125 ± 0.061	1.087 ± 0.207	0.831 ± 0.043	0.902 ± 0.087	0.915 ± 0.063	1.048 ± 0.131	0.984 ± 0.085
EF (%)	63.039 ± 3.293	66.715 ± 14.514	69.349 ± 2.825	70.307 ± 3.332	55.577 ± 1.167	73.970 ± 4.672	57.587 ± 3.187	77.095 ± 5.965	60.496 ± 7.564
FS (%)	33.532 ± 2.489	36.732 ± 12.009	38.555 ± 2.224	40.432 ± 2.42	28.027 ± 0.785	42.664 ± 3.891	29.735 ± 2.036	47.753 ± 5.779	31.656 ± 10.350
LV Mass (mg)	53.316 ± 3.788	60.809 ± 24.377	92.076 ± 2.407	84.784	59.599 ± 6.413	49.695 ± 5.528	72.767 ± 5.716	67.939 ± 6.024	65.085 ± 8.819
LV Vol; d (mm^3)	39.041 ± 3.031	33.060 ± 12.916	53.657 ± 1.887	48.401 ± 3.668	40.943 ± 3.405	30.247 ± 3.869	47.989 ± 5.184	41.480 ± 6.584	43.323 ± 6.454
LV Vol; s (mm^3)	14.530 ± 1.917	12.024 ± 9.106	16.601 ± 1.833	13.98 ± 1.954	18.157 ± 1.613	8.963 ± 2.727	20.358 ± 2.722	11.851 ± 4.177	17.124 ± 2.067
HR (beats/min)	427 ± 20	447 ± 13	502.548 ± 23	493.224 ± 13.506	456 ±17	431 ± 24	440 ± 18	432 ± 16	458 ± 22
HW (mg) / BW (g)	8.032 ± 0.182	8.294 ± 0.247	7.076 ± 0.231	6.845 ± 0.278	8.242 ± 0.182	8.054 ± 0.399	8.163 ± 0.190	9.002 ± 0.265	8.703 ± 0.183

Data are means ± SEM (n=8). Student's t-test was used for two-sample comparisons and two-way ANOVA with Tukey post-hoc tests for comparisons between multiple groups when there were two experimental factors. IVS, inerventricular septum; LVID, left ventricle internal dimension; LVPW, left ventricle posterior wall; EF, ejection fraction; FS, fractional shortening; LV Mass, left ventricle mass; LV Vol, left ventricle volume; HR, heart rate; HW, heart weight; BW, body weight.

Table S2: Echocardiology and weight analysis of adult myocardial infarction model

Echocardiology Data (MId28)	WT	Foxp1^{CMKO}	Foxp1^{CMTg}	WT + Hand1-AAV9	Foxp1^{CMTg} + Hand1-AAV9
IVS; d (mm)	0.597 ± 0.044	0.658 ± 0.043	0.556 ± 0.075	0.737 ± 0.055	0.595 ± 0.059
IVS; s (mm)	0.804 ± 0.078	0.938 ± 0.085*	0.668 ± 0.087	1.080 ± 0.129	0.791 ± 0.088
LVID; d (mm)	5.170 ± 0.206	4.601 ± 0.290	5.491 ± 0.257	4.772 ± 0.312	4.821 ± 0.297
LVID; s (mm)	4.426 ± 0.252	3.562 ± 0.307*	4.952 ± 0.286	3.598 ± 0.259*	3.976 ± 0.276$^{\#}$
LVPW; d (mm)	0.661 ± 0.070	0.700 ± 0.054	0.651 ± 0.074	0.831 ± 0.084	0.683 ± 0.064
LVPW; s (mm)	0.788 ± 0.078	0.936 ± 0.062	0.694 ± 0.094	1.191 ± 0.133*	0.910 ± 0.093
EF (%)	31.279 ± 3.217	46.035 ± 4.699*	20.770 ± 3.869*	48.924 ± 2.345*	36.918 ± 2.511$^{\#\#}$
FS (%)	15.088 ± 1.660	23.277 ± 2.741*	9.708 ± 1.893*	24.786 ± 1.388*	17.823 ± 1.321$^{\#\#}$
LV Mass	131.18 ± 9.850	122.047 ± 12.483	144.966 ± 23.754	164.960 ± 31.930	121.498 ± 13.849
LV Vol; d	132.123 ± 13.177	101.337 ± 13.621	149.908 ± 17.060	110.678 ± 16.695	112.74± 14.646
LV Vol; s	95.326 ± 14.054	57.342 ± 10.768*	121.268 ± 18.320	57.449 ± 9.484*	72.510 ± 10.710$^{\#}$
HR (beats/min)	433 ± 25	427 ± 18	428 ± 21	408 ± 16	424 ± 26
HW (mg) / BW (g)	7.389 ± 2.612	6.982 ± 0.415	7.963 ± 0.640	8.063 ± 0.584	8.455 ± 0.721

Data are means ± SEM (n=8). * $P<0.05$, ** $P<0.01$, compared with wild-type group. $^{\#}P<0.05$, $^{\#\#}P<0.01$, compared with Foxp1^{CMTg} group. Student's t-test was used for two-sample comparisons and two-way ANOVA with Tukey post-hoc tests for comparisons between multiple groups when there were two experimental factors. IVS, inerventricular septum; LVID, left ventricle internal dimension; LVPW, left ventricle posterior wall; EF, ejection fraction; FS, fractional shortening; LV Mass, left ventricle mass; LV Vol, left ventricle volume; HR, heart rate; HW, heart weight; BW, body weight.

Table S3: Primer sequences for reverse transcript quantitative PCR (RT-qPCR)

Gene Name	Primer Sequence
Acsl1-mus	Forward: 5'-TGCCAGAGCTGATTGACATTC-3'<br>Reverse: 5'-GCCTCACACTCGCTGATCTT-3'
Ech1-mus	Forward: 5'-TTCTGCCTGTGACATTCGCT-3'<br>Reverse: 5'-GCAGAGAAGGTCAGCTCGTT-3'
Fabp3-mus	Forward: 5'-GTGACAGCAGATGACCGGAA-3'<br>Reverse: 5'-CTCACCACACTGCCATGAGT-3'
Foxp1-mus	Forward: 5'-AAGCAGCTAACACTAAACGAAATC-3'<br>Reverse: 5'-TTCCACGTGGCTGCATT-3'
Gapdh-mus	Forward:5'-AAATGGTGAAGGTCGGTGTGAACG-3'<br>Reverse: 5'-ATCTCCACTTTGCCACTGC-3'
Glut1-mus	Forward: 5'-CAGTTCGGCTATAACACTGGTG-3'<br>Reverse: 5'-GCCCCCGACAGAGAAGATG-3'
Hand1-mus	Forward: 5'-AAGATCAAGACTCTGCGCCT-3'<br>Reverse: 5'-CAGGAGGGAAGCTTTCGGG-3'
Hif1a-mus	Forward: 5'-CGCCTCTGGACTTGTCTCTT-3'<br>Reverse: 5'-TCGACGTTCAGAACTCATCCT-3'
Hk2-mus	Forward: 5'-TGATCGCCTGCTTATTCACGG-3'<br>Reverse: 5'-AACCGCCTAGAAATCTCCAGA-3'
Hmgcs2-mus	Forward:5'-CAGAAATCCCTGGCTCGGTT-3'<br>Reverse:5'-TTGAACATGTCCAGGGAGGC-3'
Hsl-mus	Forward: 5'-CCAGCCTGAGGGCTTACTG-3'<br>Reverse: 5'-CTCCATTGACTGTGACATCTCG-3'
Ldha-mus	Forward: 5'-TGTCTCCAGCAAAGACTACTGT-3'<br>Reverse: 5'-GACTGTACTTGACAATGTTGGGA-3'
Mlycd-mus	Forward: 5'-GCACGTCCGGGAAATGAAC-3'<br>Reverse: 5'-GCCTCACACTCGCTGATCTT-3'
Pdk-1-mus	Forward: 5'-GGACTTCGGGTCAGTGAATGC-3'<br>Reverse: 5'-TCCTGAGAAGATTGTCGGGGA-3'
Usp20-mus	Forward: 5'-GCTAGCTTGACTGACAGGCG-3'<br>Reverse: 5'-CTATGCAGTCCAGGTGAGGG-3'
Foxp1-homo	Forward: 5'-CTCCTCCTCTGCACCTTC-3'<br>Reverse: 5'-TGCTCCTCATGGGACAAA-3'
Gapdh-homo	Forward: 5'-ATGGAAATCCCATCACCATCTT-3'<br>Reverse:5'-CGCCCCACTTGATTTTGG-3'

Table S4: Sequences of gene-siRNA

Gene Name	siRNA sequence
mFoxp1	Forward: 5'-GCAGGUUGUACAGCAGUUATT-3'
	Reverse: 5'-UAACUGCUGUACAACCUGCTT-3'
mHif1a	Forward: 5'-CCAGUUACGUUCCUUUGAUTT-3'
	Reverse: 5'-AUCAAAGGAACGUAACUGGTT-3'
mHand1	Forward: 5'-CCAAGCUCUCCAAGAUCAATT-3'
	Reverse: 5'-UUGAUCUUGGAGAGCUUGGTT-3'
Scramble	Forward:5'-UUCUCCGAACGUGUCACGUTT-3'
	Reverse: 5'-ACGUGACACGUUCGGAGAATT-3'

Table S5: Primer sequence for qPCR of chromatin immunoprecipitation (ChIP) assays

Promoter	siRNA sequence
mUsp20	Forward: 5'-GCAAAAGAAACCAAACCACA-3'
	Reverse: 5'-AGGAGCACTGTCCACAGGAG-3'
mHand1	Forward: 5'-TGTCTGTCTGTCCGTCTGTG-3'
	Reverse: 5'-GCTCAGTGGTAGAGCACTTG-3'
mGapdh	Forward: 5'-TCCCGAGTGTTGGGATTAAA-3'
	Reverse: 5'-AATAGTTTATGCCTGGGGCC-3'

Supporting Figure Legends

Figure S1. High efficacy of Foxp1 deletion in cardiomyocytes Foxp1 deletion mice and Foxp1 loss of function enhanced post-injury cardiomyocyte proliferation. A-B, Mouse neonatal cardiomyocytes (NMCMs) were isolated, flow cytometry (A) and cell immunofluorescence staining (B) demonstrated that over 90% isolated cells were cTNT positive cardiomyocytes. **C-D,** Foxp1 expression in isolated mouse neonatal cardiomyocytes (NMCMs) of CMs specific Foxp1 deletion mice ($Foxp1^{CMKO}$) and wild-type littermates by western blot (C), RT-qPCR (D) (n=5). **E,** Foxp1 co-immunostaining with CMs marker α-sarcomeric actinin (α-SA) in heart sections of $Foxp1^{CMKO}$ mice and wild-type littermates (n=5). **F,** Gross morphology as cardiac size and structure at 12 weeks in $Foxp1^{CMKO}$ mice and wild-type littermates by hematoxylin-eosin (HE) staining after postnatal tamoxifen induction (40 μg/kg per day from postnatal day 0 to 3). **G-I,** CM proliferation quantified by EdU incorporation (G) and co-immunostaining of Ki67 (H), PH3 (I) with cardiac nuclear specific pericentriolar material 1 (pcm1) in P3ARd7 hearts of $Foxp1^{CMKO}$ mice and wild-type littermates (n=8). Data are means ± SEM. *P<0.05; ** P<0.01, n.s. indicates not significant. Scale bar: **B, E, G** though **I,** 50 μm; **F**, 2 mm.

Figure S2. Foxp1-siRNA knockdown enhanced neonatal mouse cardiomyocytes proliferation. A-B, Foxp1 expression in Foxp1-siRNA knockdown NMCMs isolated from postnatal day 1-3 (P1-P3) mice and by western blot (A) and RT-qPCR (B) (n=5). **C-E,** EdU incorporation (C) and immunostaining of pH3 (D), Aurora B (E) in $\alpha\text{-SA}^+$ staining CMs of Foxp1-siRNA knockdown and scramble-siRNA control (n=5). **F-G,** Cell cycle genes expression in Foxp1-siRNA knockdown and scramble-siRNA treated NMCMs were detected by RT-qPCR (F) and western blot (G) (n=5). Data are means ± SEM. *P<0.05; ** P<0.01, n.s. indicates not significant. Scale bar: **C** though **E**, 50 μm.

Figure S3. Loss of Foxp1 in cardiomyocytes increases cell proliferation in adult cardiomyocytes and promote heart regeneration after adult mouse myocardial infarction injury. A-B, Foxp1 expression in isolated mouse adult CMs of $Foxp1^{CMKO}$ mice and wild-type littermates by western blot (A) and RT-qPCR (B) (n=5). **C,** Evans blue dye perfusion of hearts and Triphenyl tetrazolium chloride (TTC) staining and quantification of area at risk (AAR) and infarct size (IS) / AAR ratio in cardiac sections 1 day post myocardial infarction (MId1) of $Foxp1^{CMKO}$ mice and wild-type littermates (n=5). **D-E,** Cumulative survival curve (Kaplan-Meier survival plot) (D) and serial echocardiographic measurements of ejection fraction (E) for long term analysis of survival and cardiac function in $Foxp1^{CMKO}$ mice and wild-type littermates.

F, Border zone CMs isolated at MI28 hearts from Foxp1^{CMKO} and wild-type littermates for evaluation of the percentage of mono-nucleated, bi-nucleated and multi-nucleated CMs (n=5). **G-I,** Border zone CM proliferation quantified by Edu incorporation (G), co-immunostaining of Ki67 (H), PH3 (I) with pericentriolar material 1 (pcm1) in MId14 hearts from Foxp1^{CMKO} mice and wild-type littermates. **J-M**, Human iPSC-CMs were transfected with Foxp1-siRNA or scramble (sc)-siRNA, Foxp1 efficacy were detected by RT-qPCR (J) and western blot (K). Cell proliferation was quantified by Edu incorporation (L) and co-immunostaining of PH3 (M) in α-SA$^+$ staining iPSC-CMs of Foxp1-siRNA knockdown and sc-siRNA control (n=5). Data are means ± SEM. *P<0.05; ** P<0.01, n.s. indicates not significant. Scale bar: **C**, 2 mm ; **F**, 10 μm; **G** through **I** and **L**, **M**, 50 μm.

Figure S4. Cardiomyocytes Foxp1 induced expression diminishes cardiomyocytes proliferation and impairs in vitro neonatal cardiac regeneration. A, Schematic diagram for generation of CMs specific Foxp1 gain-of-function mouse (Foxp1^{CMTg}). **B-C,** Foxp1 expression in NMCMs of Foxp1^{CMTg} and wild-type mice by western blot (B) and by RT-qPCR (C) (n=5). Data are means ± SEM. ** P<0.01, n.s. indicates not significant.

Figure S5. Generation of mice with Hif1α and Foxp1 double deletion in cardiomyocytes and Hif1α knockdown in cultured NMCMs reverses the enhanced NMCMs proliferation upon Foxp1 deletion. A, Schematic diagram for generation of CMs specific Hif1α (Hif1α^{CMKO}) and Hif1α;Foxp1 (Hif1α; Foxp1^{CMKO}) double deletion mice. **B-C,** Hif1α protein expression by western blots in P3ARd3 or sham operated CMs of Hif1α^{CMKO} mice and wild-type littermates (B), and Hif1α transcriptional expression by RT-qPCR in P6 CMs of Hif1α^{CMKO} mice and wild-type littermates (C) (n=5). **D-E,** Hif1α expression of P1-P3 NMCMs with Hif1α-siRNA knockdown and scramble-siRNA control by western blot (D) and RT-qPCR (E) (n=5). **F-H,** Edu incorporation (F) and immunostaining of pH3 (G), Aurora B (H) in α-SA$^+$ staining CMs of Hif1α-siRNA, Foxp1-siRNA and Hif1α-siRNA/Foxp1-siRNA knockdown compared to the scramble-siRNA control (n=4). Data are means ± SEM. *P<0.05; ** P<0.01, n.s. indicates not significant. Scale bar: **F** though **H,** 50 μm.

Figure S6. Foxp1 knockdown increases glucose utilization and lactate production of NMCMs whereas Hif1α or Hand1 knockdown reverses its elevated glucose utilization and lactate production. A-C, Glucose utilization and lactate production in NMCMs of Foxp1-siRNA knockdown (A), NMCMs of Hif1α-siRNA, Foxp1-siRNA or Hif1α-siRNA/Foxp1-siRNA knockdown (B), and NMCMs of Hand1-siRNA, Foxp1-siRNA or Hand1-

siRNA/Foxp1-siRNA knockdown (C) compared to the scramble-siRNA control (n=5). Data are means ± SEM. **P*<0.05; ** *P*<0.01. n.s. indicates not significant.

Figure S7. Induced Hand1 expression in cardiomyocytes by cTnT promoter-driven adeno-associated virus 9 significantly enhances NMCMs proliferation *in vitro*. A, Schematic diagram of construction of Hand1 overexpression in CMs by cTnT promoter driven AAV9 (AAV9-cTnTp-Hand1- EGFP). **B-D,** Hand1 expression in NMCMs infected with AAV9-cTnTp-Hand1-EGFP by Fluorescent microscopy and bright filed images (A), and western blot (C) and RT-qPCR (D) (n=5). **E-G,** EdU incorporation (E) and immunostaining of PH3 (F), Aurora B (G) in α-SA^+ staining CMs of Foxp1 gain-of-function and Hand1 overexpression (n=5). **H-I**, Human iPSCs-CMs were infected with AAV9-cTnTp-Hand1-EGFP or scramble AAV9 vector, Edu incorporation (H) and co-immunostaining of PH3 (I) with α-SA^+ for cell proliferation detection. Data are means ± SEM. **P*<0.05; ** *P*<0.01. Scale bar: **B**, **E** though **G,** 50 μm.

Figure S8. cTnT promoter driven AAV9 have high efficacy of Hand1 induced expression in mouse cardiomyocytes *in vivo*. A-C, AAV9-cTnTp-Hand1-EGFP colocalized with CMs specific marker cTnT (Red) in mouse hearts 14 days following viral injection (A), and Hand1 expression in isolated CMs shown by western blot (B) and RT-qPCR (C) (n=5). Data are means ± SEM. ** *P*<0.01. Scale bar: **A,** 50 μm.

Figure S9. Working model of cardiomyocyte-specific Foxp1 regulation of postinjury heart regeneration through USP20-Hif1α signal pathway. The analysis of datasets from previous single-cell transcriptome studies of regenerative hearts shows a significant reduction of Foxp1 expression in the highly proliferative capacity CMs at MI border zone. The further studies of both CM-specific Foxp1 loss- and gain-of function mice demonstrate the beneficial effects of Foxp1 deletion in CMs on metabolic reprogram from FA oxidation to glycolysis to improve CMs proliferation for heart regeneration and repair, while Foxp1 induced expression in CMs has the opposite effects. We identified the USP20-Hif1α-Hand1 transcription network as a Foxp1 direct downstream target genes for the regulation of CM proliferation which controlling the heart regeneration for injury repair and dysfunction recovery. "Created in BioRender. Wu, W. (2024) https://BioRender.com/d06l610 ".

References

[1] M. Cui, Z. Wang, K. Chen, A. M. Shah, W. Tan, L. Duan, E. Sanchez-Ortiz, H. Li, L. Xu, N. Liu, R. Bassel-Duby, E. N. Olson, *Dev Cell* **2020**, *55* (5), 665.
[2] A. I. Mahmoud, E. R. Porrello, W. Kimura, E. N. Olson, H. A. Sadek, *Nat Protoc* **2014**, *9* (2), 305.
[3] E. R. Porrello, A. I. Mahmoud, E. Simpson, J. A. Hill, J. A. Richardson, E. N. Olson, H. A. Sadek, *Science* **2011**, *331* (6020), 1078.
[4] Y. Kuang, X. Li, X. Liu, L. Wei, X. Chen, J. Liu, T. Zhuang, J. Pi, Y. Wang, C. Zhu, X. Gong, H. Hu, Z. Yu, J. Li, P. Yu, H. Fan, Y. Zhang, Z. Liu, L. Zhang, *Cardiovasc Res* **2021**, *117* (2), 585.
[5] Y. Wu, L. Zhou, H. Liu, R. Duan, H. Zhou, F. Zhang, X. He, D. Lu, K. Xiong, M. Xiong, J. Zhuang, Y. Liu, L. Li, D. Liang, Y. H. Chen, *Cell Res* **2021**, *31* (4), 450.
[6] S. Gao, D. Ho, D. E. Vatner, S. F. Vatner, *Curr Protoc Mouse Biol* **2011**, *1*, 71.
[7] J. Liu, T. Zhuang, J. Pi, X. Chen, Q. Zhang, Y. Li, H. Wang, Y. Shen, B. Tomlinson, P. Chan, Z. Yu, Y. Cheng, X. Zheng, M. Reilly, E. Morrisey, L. Zhang, Z. Liu, Y. Zhang, *Circulation* **2019**, *140* (8), 665.
[8] W. Yu, X. Huang, X. Tian, H. Zhang, L. He, Y. Wang, Y. Nie, S. Hu, Z. Lin, B. Zhou, W. Pu, K. O. Lui, B. Zhou, *Development* **2016**, *143* (6), 936.
[9] M. M. Lu, S. Li, H. Yang, E. E. Morrisey, *Gene Expr Patterns* **2002**, *2* (3-4), 223.
[10] S. Bohl, D. J. Medway, J. Schulz-Menger, J. E. Schneider, S. Neubauer, C. A. Lygate, *Am J Physiol Heart Circ Physiol* **2009**, *297* (6), H2054.
[11] M. Ackers-Johnson, P. Y. Li, A. P. Holmes, S. M. O'Brien, D. Pavlovic, R. S. Foo, *Circ Res* **2016**, *119* (8), 909.
[12] Y. Tian, Y. Zhang, L. Hurd, S. Hannenhalli, F. Liu, M. M. Lu, E. E. Morrisey, *Development* **2011**, *138* (7), 1235.

Figure S1

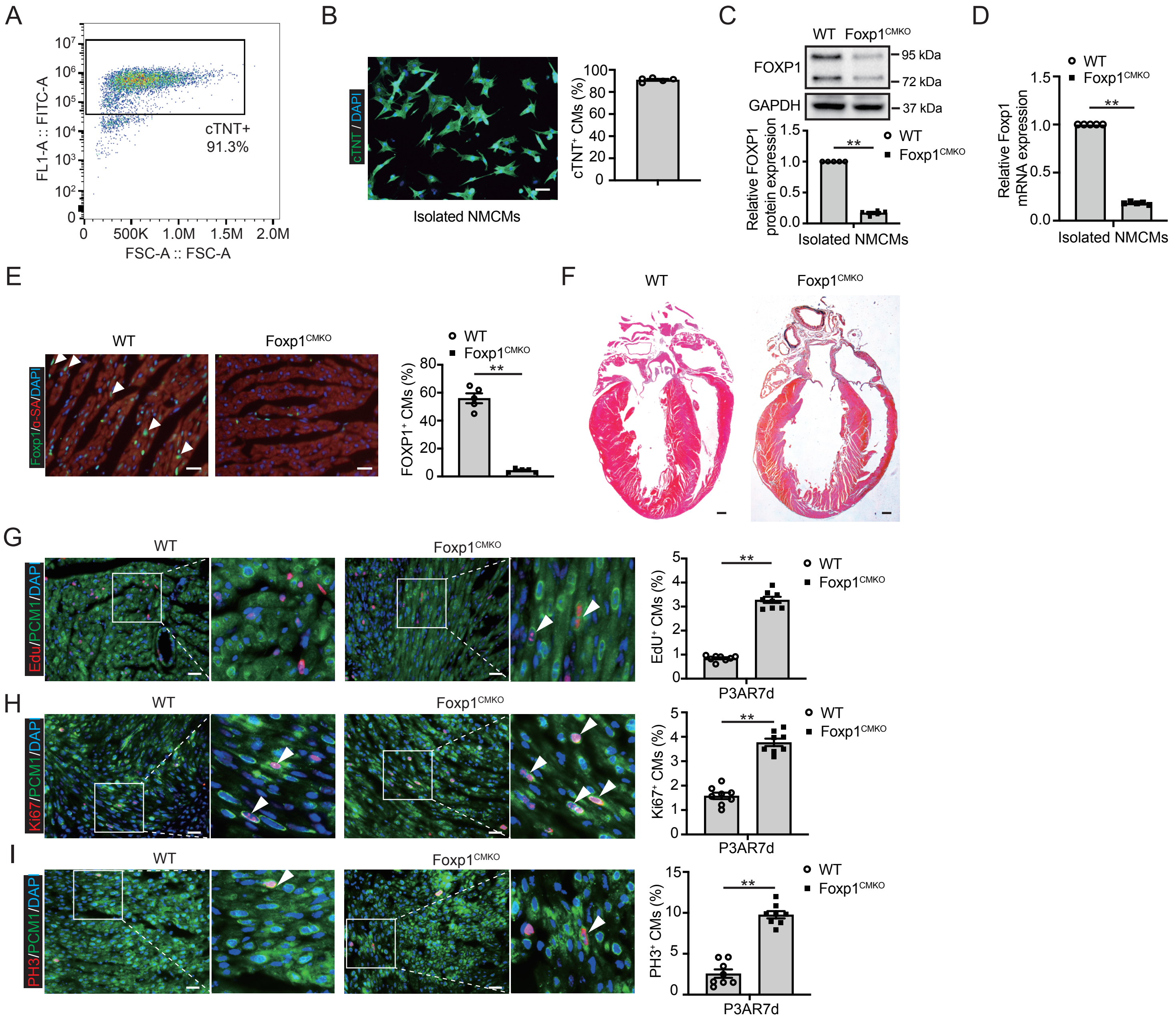

A
FL1-A :: FITC-A
FSC-A :: FSC-A
cTNT+
91.3%
B
cTNT / DAPI
Isolated NMCMs
cTNT+ CMs (%)
C
WT Foxp1CMKO
FOXP1
95 kDa
72 kDa
GAPDH
37 kDa
Relative FOXP1 protein expression
Isolated NMCMs
D
Relative Foxp1 mRNA expression
Isolated NMCMs
E
WT
Foxp1CMKO
Foxp1/α-SA/DAPI
FOXP1+ CMs (%)
F
WT
Foxp1CMKO
G
Edu/PCM1/DAPI
EdU+ CMs (%)
P3AR7d
H
Ki67/PCM1/DAPI
Ki67+ CMs (%)
P3AR7d
I
PH3/PCM1/DAPI
PH3+ CMs (%)
P3AR7d

Figure S2

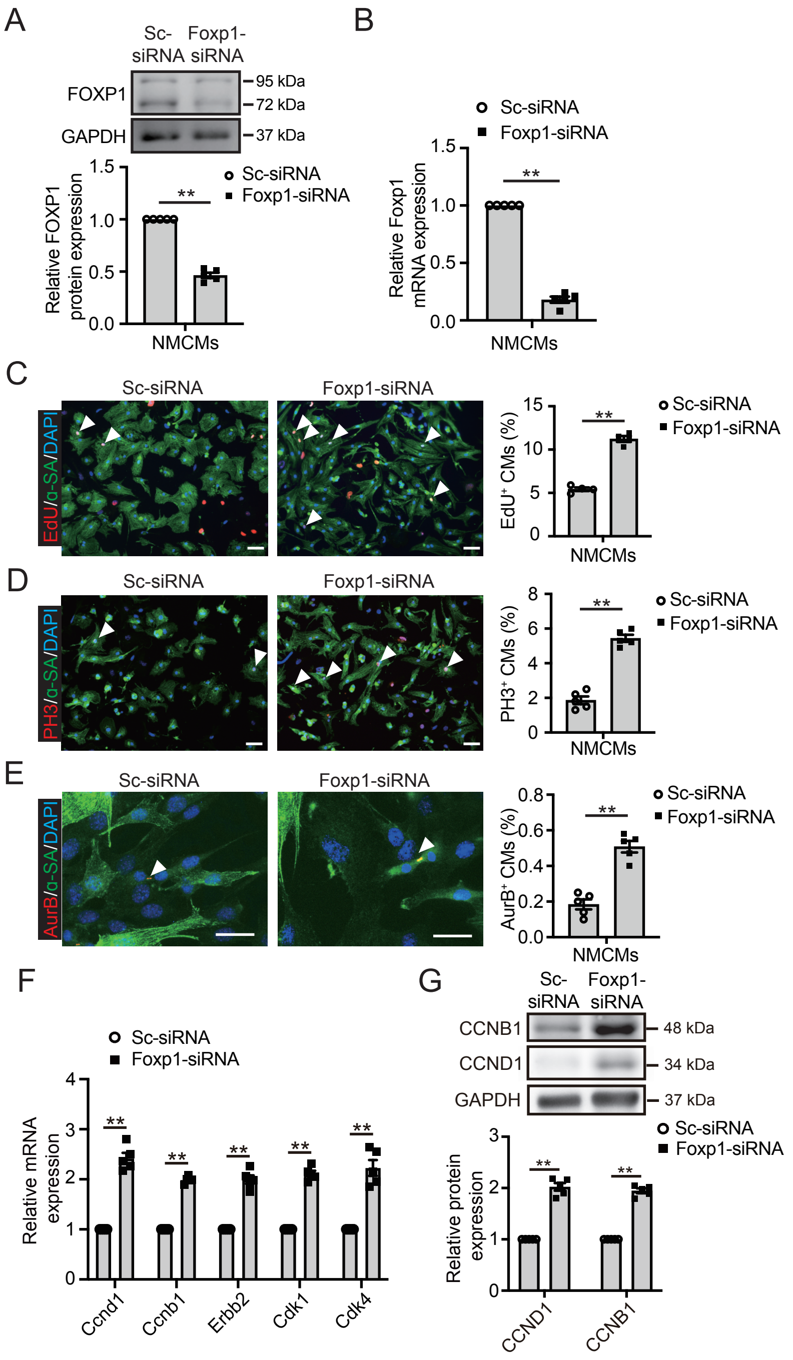
A
Sc-siRNA
Foxp1-siRNA
FOXP1
GAPDH
95 kDa
72 kDa
37 kDa
Relative FOXP1 protein expression
NMCMs
B
Relative Foxp1 mRNA expression
C
EdU/α-SA/DAPI
EdU⁺ CMs (%)
D
PH3/α-SA/DAPI
PH3⁺ CMs (%)
E
AurB/α-SA/DAPI
AurB⁺ CMs (%)
F
Relative mRNA expression
Ccnd1
Ccnb1
Erbb2
Cdk1
Cdk4
G
CCNB1
CCND1
48 kDa
34 kDa
Relative protein expression

Figure S3

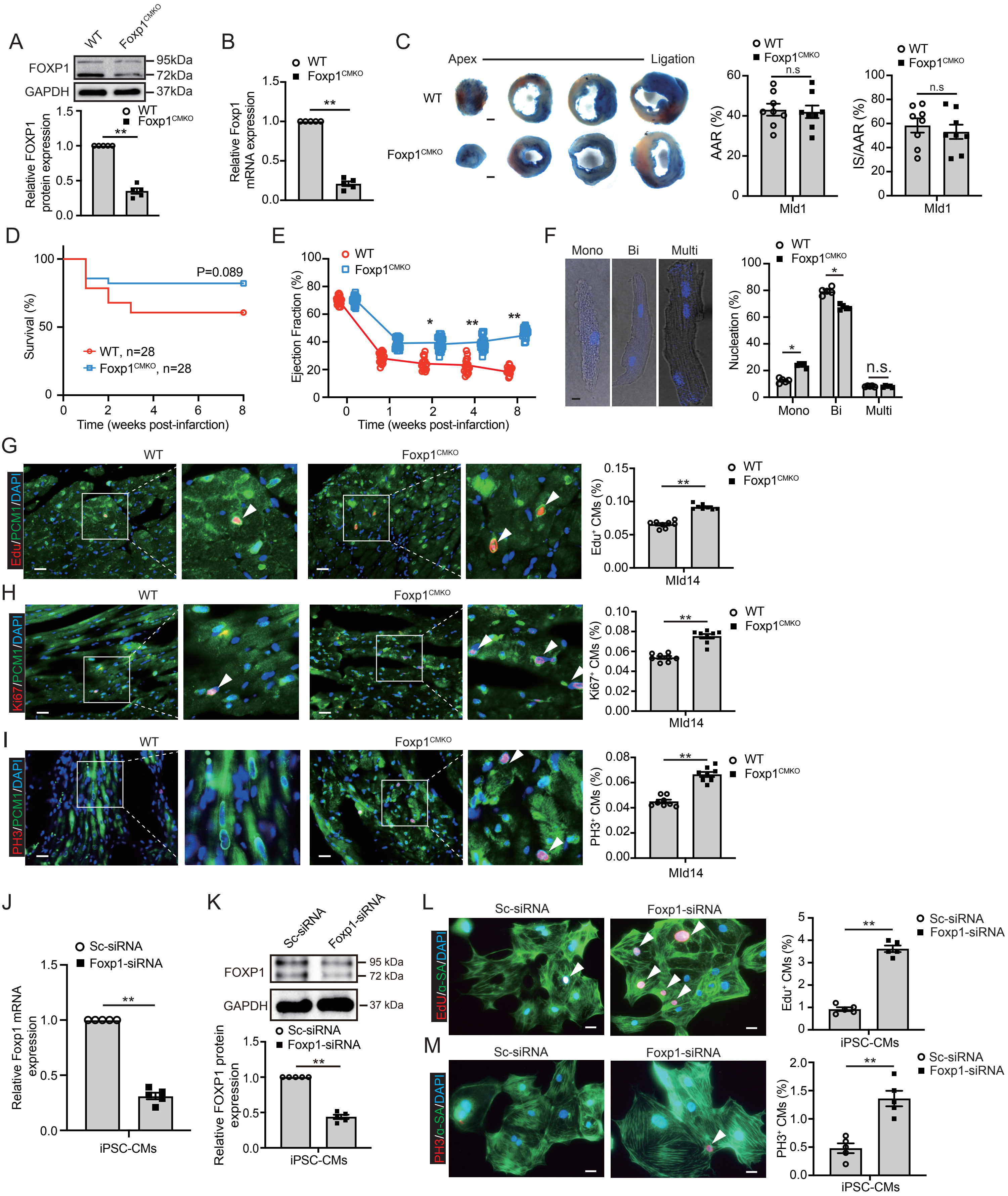

A
WT
Foxp1CMKO
FOXP1
GAPDH
95kDa
72kDa
37kDa
Relative FOXP1 protein expression
B
Relative Foxp1 mRNA expression
C
Apex
Ligation
AAR (%)
IS/AAR (%)
MId1
n.s
D
Survival (%)
P=0.089
WT, n=28
Foxp1CMKO, n=28
Time (weeks post-infarction)
E
Ejection Fraction (%)
Time (weeks post-infarction)
F
Mono
Bi
Multi
Nucleation (%)
n.s.
G
Edu/PCM1/DAPI
Edu+ CMs (%)
MId14
H
Ki67/PCM1/DAPI
Ki67+ CMs (%)
MId14
I
PH3/PCM1/DAPI
PH3+ CMs (%)
MId14
J
Sc-siRNA
Foxp1-siRNA
Relative Foxp1 mRNA expression
iPSC-CMs
K
Sc-siRNA
Foxp1-siRNA
FOXP1
GAPDH
95 kDa
72 kDa
37 kDa
Relative FOXP1 protein expression
iPSC-CMs
L
EdU/α-SA/DAPI
Edu+ CMs (%)
iPSC-CMs
M
PH3/α-SA/DAPI
PH3+ CMs (%)
iPSC-CMs

Figure S4

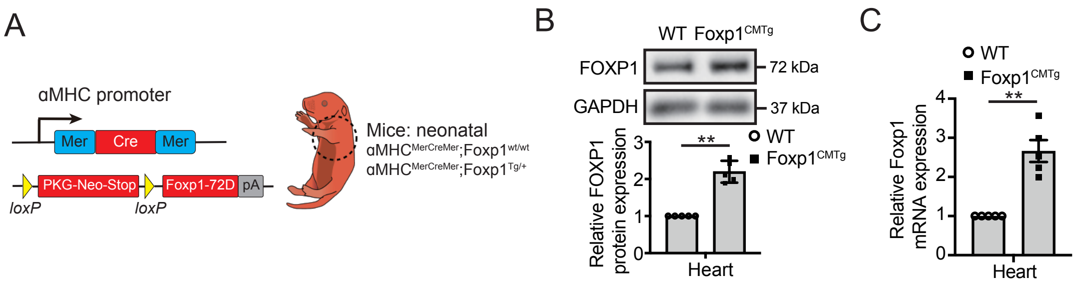
A
αMHC promoter
Mer
Cre
Mer
PKG-Neo-Stop
Foxp1-72D
pA
loxP
loxP
Mice: neonatal
αMHC^MerCreMer;Foxp1^wt/wt
αMHC^MerCreMer;Foxp1^Tg/+
B
WT
Foxp1^CMTg
FOXP1
72 kDa
GAPDH
37 kDa
Relative FOXP1 protein expression
WT
Foxp1^CMTg
**
Heart
C
WT
Foxp1^CMTg
Relative Foxp1 mRNA expression
**
Heart

Figure S5

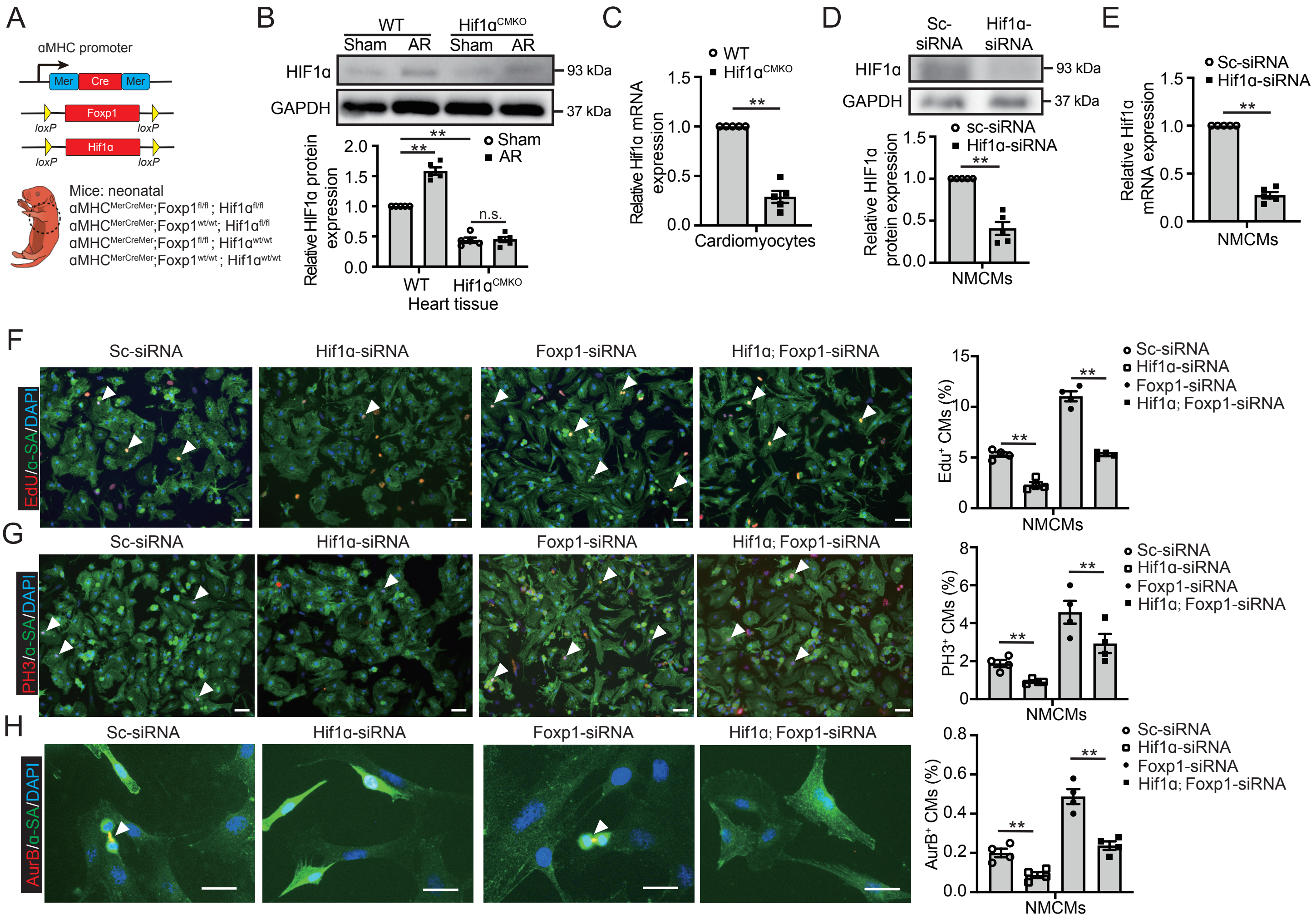
A
αMHC promoter
Mer Cre Mer
Foxp1
loxP loxP
Hif1α
loxP loxP
Mice: neonatal
αMHCMerCreMer;Foxp1fl/fl; Hif1αfl/fl
αMHCMerCreMer;Foxp1wt/wt; Hif1αfl/fl
αMHCMerCreMer;Foxp1fl/fl; Hif1αwt/wt
αMHCMerCreMer;Foxp1wt/wt; Hif1αwt/wt
B
WT Hif1αCMKO
Sham AR Sham AR
HIF1α 93 kDa
GAPDH 37 kDa
Relative HIF1α protein expression
Sham
AR
n.s.
WT Hif1αCMKO
Heart tissue
C
WT
Hif1αCMKO
Relative Hif1α mRNA expression
Cardiomyocytes
D
Sc-siRNA Hif1α-siRNA
HIF1α 93 kDa
GAPDH 37 kDa
sc-siRNA
Hif1α-siRNA
Relative HIF1α protein expression
NMCMs
E
Sc-siRNA
Hif1α-siRNA
Relative Hif1α mRNA expression
NMCMs
F
Sc-siRNA Hif1α-siRNA Foxp1-siRNA Hif1α; Foxp1-siRNA
EdU/α-SA/DAPI
Edu+ CMs (%)
NMCMs
G
PH3/α-SA/DAPI
PH3+ CMs (%)
NMCMs
H
AurB/α-SA/DAPI
AurB+ CMs (%)
NMCMs

Figure S6

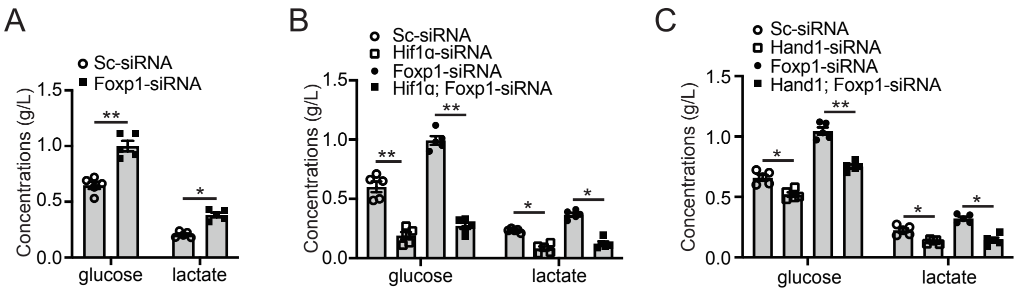
A
Sc-siRNA
Foxp1-siRNA
Concentrations (g/L)
1.5
1.0
0.5
0.0
**
*
glucose
lactate
B
Sc-siRNA
Hif1α-siRNA
Foxp1-siRNA
Hif1α; Foxp1-siRNA
Concentrations (g/L)
1.5
1.0
0.5
0.0
**
**
*
*
glucose
lactate
C
Sc-siRNA
Hand1-siRNA
Foxp1-siRNA
Hand1; Foxp1-siRNA
Concentrations (g/L)
1.5
1.0
0.5
0.0
*
**
*
*
glucose
lactate

Figure S7

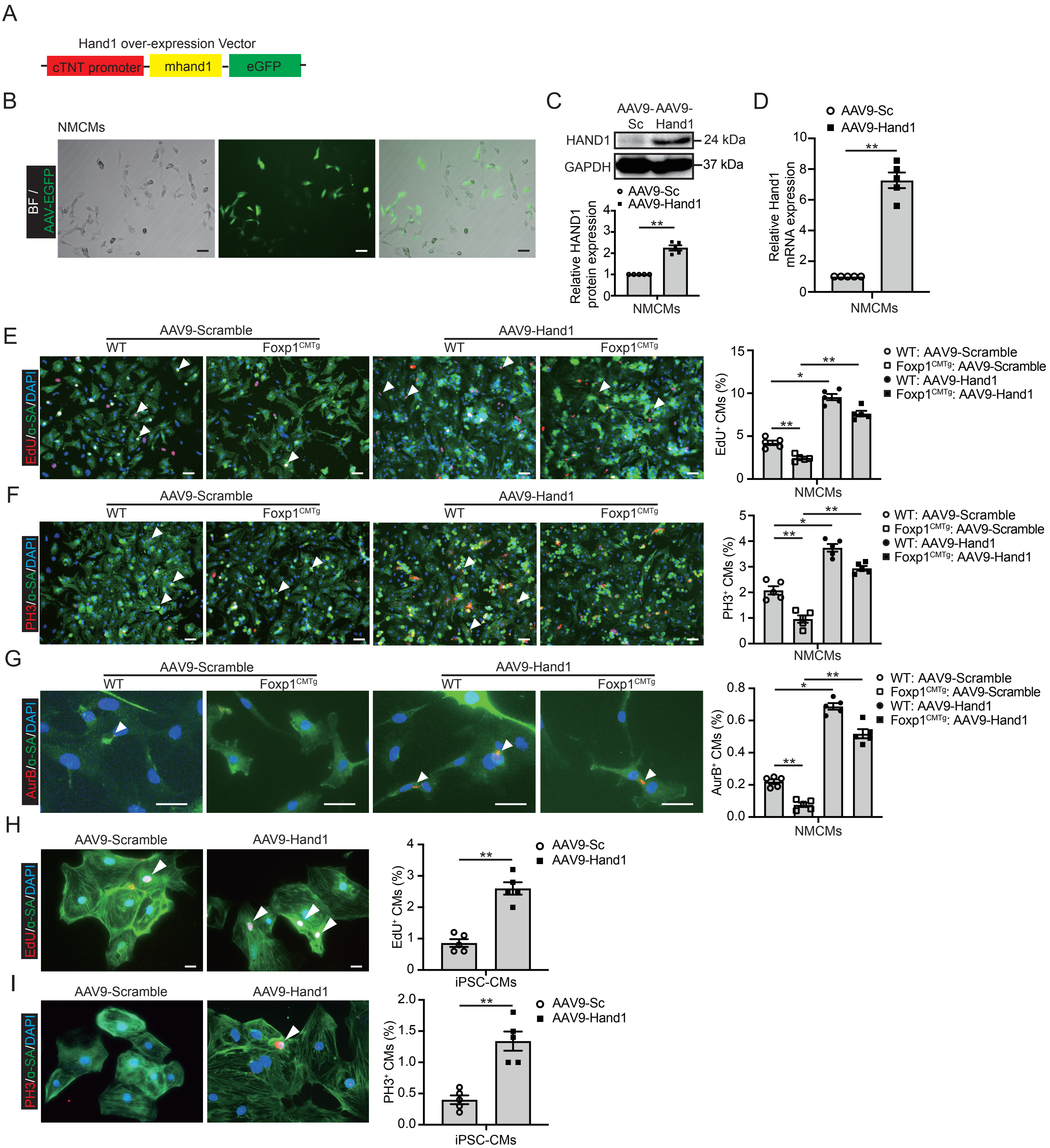
A
Hand1 over-expression Vector
cTNT promoter
mhand1
eGFP
B
NMCMs
BF / AAV-EGFP
C
AAV9-Sc
AAV9-Hand1
HAND1
24 kDa
GAPDH
37 kDa
Relative HAND1 protein expression
NMCMs
D
AAV9-Sc
AAV9-Hand1
Relative Hand1 mRNA expression
NMCMs
E
AAV9-Scramble
AAV9-Hand1
WT
Foxp1CMTg
EdU/α-SA/DAPI
EdU+ CMs (%)
WT: AAV9-Scramble
Foxp1CMTg: AAV9-Scramble
WT: AAV9-Hand1
Foxp1CMTg: AAV9-Hand1
NMCMs
F
PH3/α-SA/DAPI
PH3+ CMs (%)
G
AurB/α-SA/DAPI
AurB+ CMs (%)
H
EdU/α-SA/DAPI
EdU+ CMs (%)
iPSC-CMs
I
PH3/α-SA/DAPI
PH3+ CMs (%)
iPSC-CMs

Figure S8

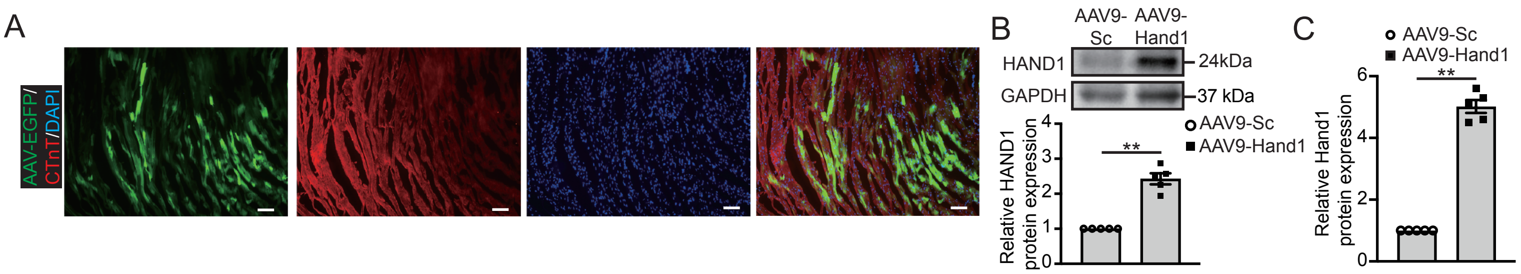
A
AAV-EGFP/CTnT/DAPI
B
AAV9-Sc
AAV9-Hand1
HAND1
24kDa
GAPDH
37 kDa
AAV9-Sc
AAV9-Hand1
Relative HAND1 protein expression
**
C
AAV9-Sc
AAV9-Hand1
Relative Hand1 protein expression
**

Figure S9

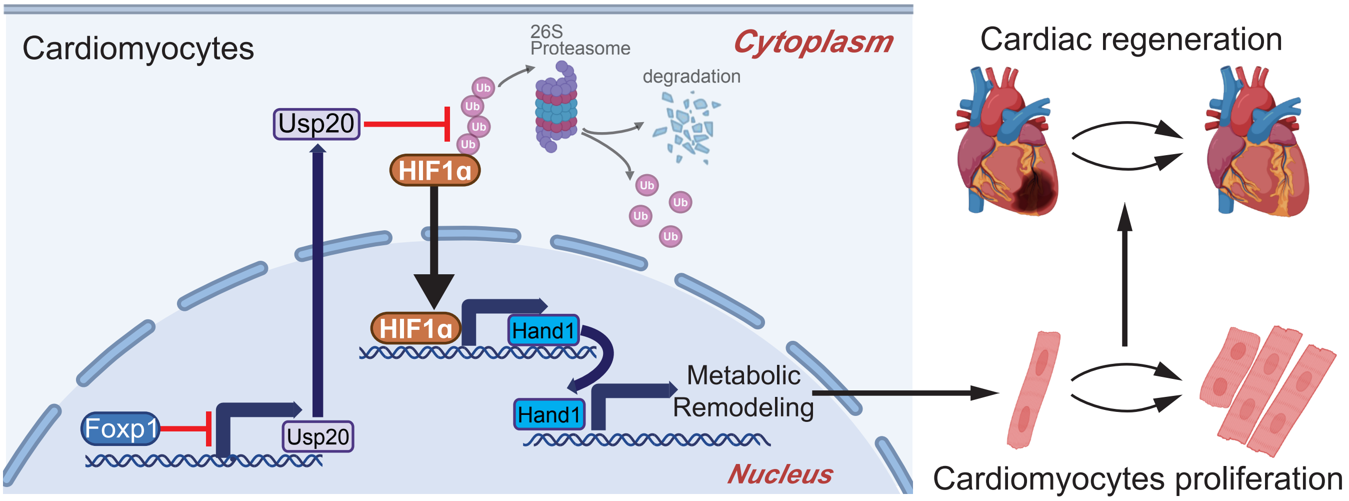

Cardiomyocytes
Cytoplasm
26S
Proteasome
degradation
Usp20
HIF1α
Ub
HIF1α
Hand1
Hand1
Metabolic
Remodeling
Foxp1
Usp20
Nucleus
Cardiac regeneration
Cardiomyocytes proliferation
